# Translation of ceragenin affinity for bacteria to an imaging reagent for infection†

**DOI:** 10.1039/c9ra02226k

**Published:** 2019-05-08

**Authors:** Nilantha Bandara, Yubo Li, Philipp Diebolder, Cedric Mpoy, Xiaobo Gu, Pitambar Khanal, Shenglou Deng, Buck E. Rogers, Paul B. Savage

**Affiliations:** Department of Radiation Oncology, Washington University School of Medicine St. Louis MO 63108 USA; Brigham Young University, Department of Chemistry and Biochemistry Provo UT 84602 USA paul_savage@byu.edu

## Abstract

Responses to bacterial infections may be manifest systemically without evidence of the location of the infection site. A rapid means of pinpointing infection sites would be useful in providing effective and possibly localized treatment. Successful means of identifying infection sites would require two components: (1) a molecule capable of recognizing bacteria and (2) a means of communicating recognition. For the recognition element, we used a ceragenin, a small molecule with affinity for bacterial membranes that was designed as a mimic of endogenous antimicrobial peptides. For the communication element, we used ^64^Cu, which is a positron emitter. By conjugating a copper chelating group to the ceragenin, the two elements were combined. Chelation of ^64^Cu by the conjugate was effective and provided a stable complex that allowed *in vivo* imaging. When administered to mice in a thigh infection model, the ^64^Cu-labeled conjugate accumulated at the site of infection (right thigh) without accumulation at the complementary site (left thigh). This conjugate may provide a means of identifying infection sites in patients presenting general signs of infection without localized symptoms.

## Abstract 2

Infection imaged *via* autoradiography with ceragenin conjugated to a copper radiolabel.

## Graphical abstract

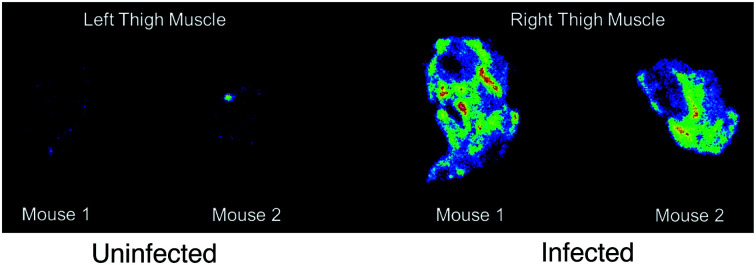

## Introduction

Identification of sites of bacterial infection is important for proper clinical care of patients manifesting general signs of infection (*e.g.*, fever and elevated white blood cell counts). This is especially true with patients that are immunocompromised because they often have reduced symptoms of infection and consequently it may be difficult to pinpoint these sites.^1,2^ Signs of inflammation can be used to identify sites of infection at superficial sites or in the extremities; however, infection in internal structures (*i.e.*, abdomen, chest and brain) can be difficult to localize at early stages.^3^ Only after infection has progressed to tissue necrosis and/or abscess formation can it be observed *via* X-ray computed tomography and ultrasound. Consequently, a method of imaging infections at their initial stages would be useful for diagnosis and treatment.

A key element for imaging infections is a targeting motif in a molecule that provides for selective association with bacteria. Multiple small molecules are known to associate with bacteria; for example, polymyxins bind the lipid A portion of lipopolysaccharides found in the outer membranes of Gram-negative bacteria.^4^ Similarly, endogenous antimicrobial peptides (AMPs) associate with Gram-negative^5^ and Gram-positive bacteria.^6^ Ceragenins are small molecule mimics of AMPs, and they also display affinity for Gram-negative and positive bacteria, specifically bacterial membrane components lipid A^6–8^ and lipotechoic acid.^6^ Due to the relatively simple structures of ceragenins, they can be readily modified, and one such modification has allowed them to be attached to silver nanoparticles.^9,10^ Silver nanoparticles, coated with a ceragenin, were placed in the presence of bacteria, and confocal imaging showed the ceragenin-appended nanoparticles associated with bacteria. This association was attributed to the affinity of the appended ceragenin for the bacterial membrane components. In a related study, the association of a fluorophore-appended ceragenin with intact cells showed that the ceragenin displayed selectivity for bacterial cells over eukaryotic cells.^7^

Nuclear medicine techniques such as positron emission tomography (PET) and single photon computed tomography (SPECT) have become a primary means of visualizing tumours and inflammation due to their high levels of sensitivity, low background interference and the three-dimensionality of their images. Some SPECT agents have been developed that allow imaging of infection. For example, ^67^Ga-citrate binds to bacteria, but it does not discriminate between bacterial infections and proteins that accumulate at sites of inflammation.^11,12^ Similarly, ^99m^Tc-labeled compounds have been used to label leukocytes for infection/inflammation imaging; however, these have been shown to be non-specific for bacterial infections.^13,14^ The use of radiolabeled antibiotics such as ciprofloxacin and kanamycin has also suffered from a lack of specificity to date.^15^ Radiolabeled AMPs, such as ubiquicidin, human lactoferrin, and human β-defensin-3, also appear promising.^15^

As mimics of AMPs and due to their simplicity and insensitivity to proteases, we reasoned that ceragenins might be well suited for imaging of infections. By conjugating a ceragenin (bacteria targeting) to an appropriate metal chelating group (for complexing a positron-emitting radiometal), we generated compounds with features necessary for selectively imaging infections. In initial studies of infection imaging, ceragenins were non-specifically labelled with ^99m^Tc and successfully imaged an infection (*Staphylococcus aureus*) model in rats.^16^ However, the nature of the ceragenin–technetium complex was not well established. Additionally, ceragenins display higher cell selectivity for Gram-negative bacteria (*e.g.*, *Escherichia coli*) over Gram-positive organisms (*e.g.*, *S. aureus*). Consequently, we anticipated that Gram-negative infections would be preferentially labelled by ceragenin conjugates.

Conjugates of a ceragenin with 1,4,7-triazacyclononane-1,4,7-triacetic acid (NOTA) were prepared, along with structural variants, and these were stably labelled with ^64^Cu and injected into mice. Distribution of the labelled compounds was determined in un-infected mice and in mice with a local thigh infection. ^64^Cu-labeled conjugates derived from a ceragenin accumulated in the infected muscle in significantly greater amounts than structural variants lacking a ceragenin. The presence of a C_8_ lipid chain on a ceragenin-containing conjugate increased the lipophilicity of the compound but did not significantly alter accumulation of the conjugate in infected muscle.

## Results and discussion

**Fig. 1:**
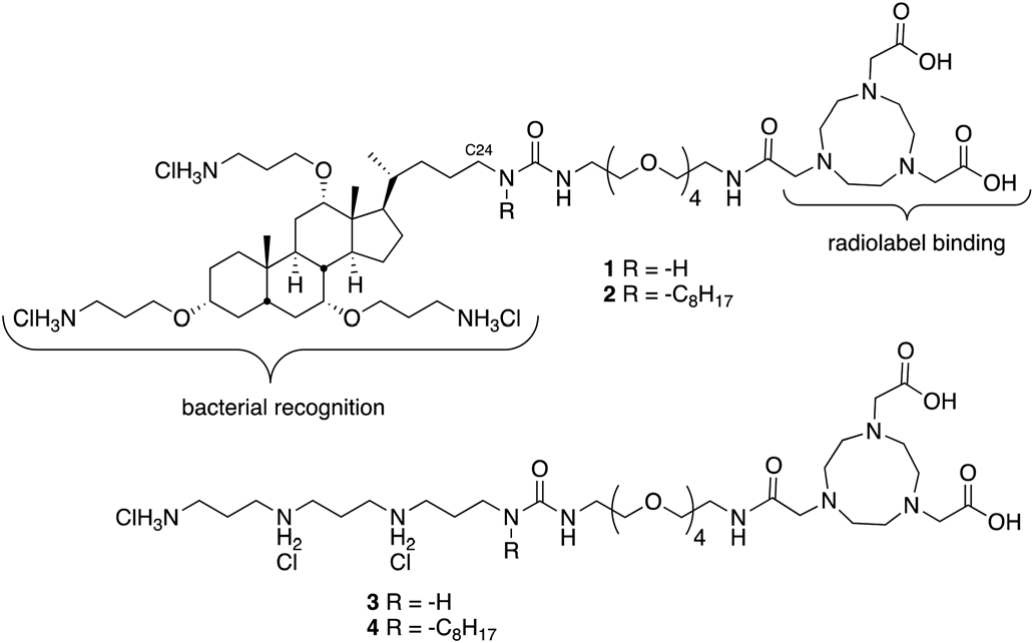
Structures of ceragenin-NOTA conjugates 1 and 2 and structural variants 3 and 4.

We have found that lipid chains extending from the nitrogen on C24 of ceragenins impact their antimicrobial properties.^17^ Consequently, two different forms of ceragenin were used as bacterial membrane recognition elements: one without a lipid chain (1) and one with an octyl chain (2) (Fig. 1). NOTA was used for binding ^64^Cu, which is a positron emitter with a 12.7 hour half-life. NOTA has been well characterized as a thermodynamically and kinetically stable copper binding ligand suitable for conjugating to a variety of targeting groups.^18^ An oligo-ethylene glycol linker was used to separate the ceragenin and NOTA to ensure that the bound copper ion did not interfere with interactions with bacterial membranes. In efforts to determine the structural features of conjugates 1 and 2 that impact bacterial labelling and imaging, structural variants 3 and 4 were prepared. Variants 3 and 4 retain three positive charges and lack the bile acid backbone found in 1 and 2. The absence (1 and 3) or presence (2 and 4) of an octyl lipid chain was used to determine its role in association with bacteria.

**Scheme 1:**
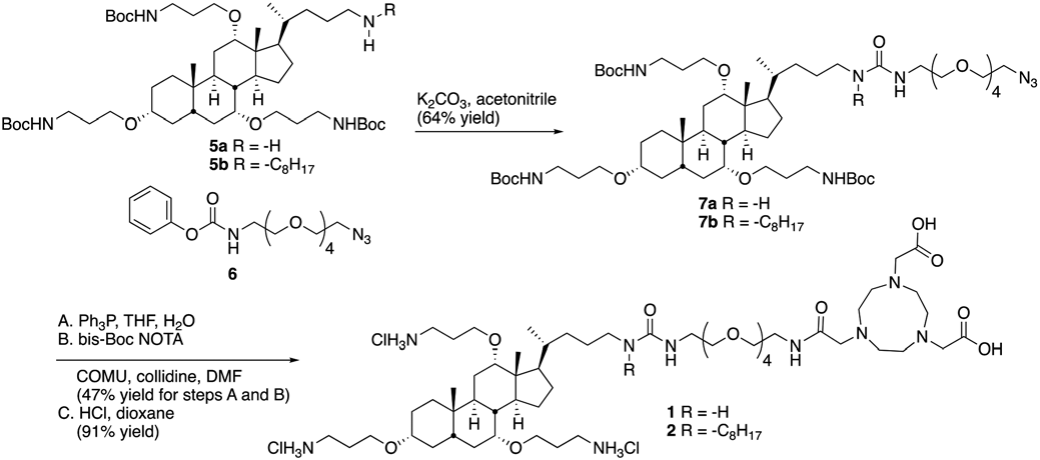
Preparation of ceragenin-NOTA conjugates 1 and 2.

To generate desired conjugates 1 and 2, amines 5a and 5b^7^ (Scheme 1) were reacted with phenylcarbamate 6 to give 7a and 7b in reasonable yields. The azide groups in 7a and 7b were reduced to the corresponding amines, which were reacted with bis-Boc NOTA, using COMU as a coupling agent. Deprotection of both the carbamates and the *t*-butyl esters with hydrogen chloride in dioxane gave 1 and 2.

**Scheme 2:**
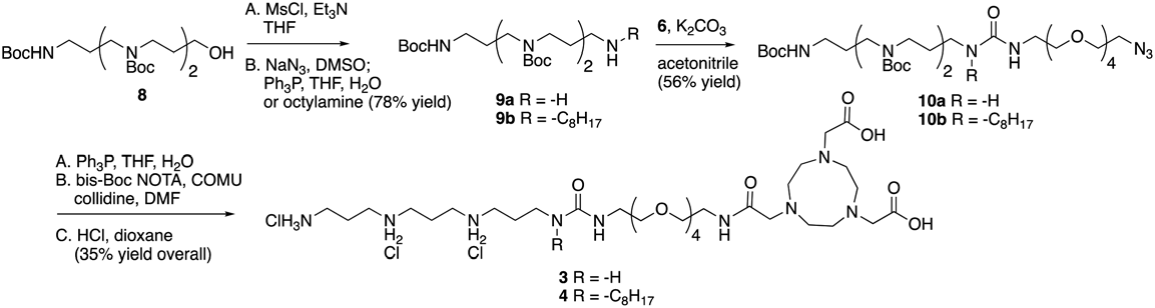
Preparation of tri-ammonium-NOTA conjugates 3 and 4.

For generation of 3 and 4 the steps outlined in Scheme 2 were followed. Alcohol in 8 (prepared using methods described in ref. 19) was converted to the mesylate and then to either the corresponding amine 9a or octylamine 9b. Coupling with the PEG tether 6 gave 10a and 10b, which were reduced and coupled with NOTA to give 3 and 4.

**Fig. 2:**
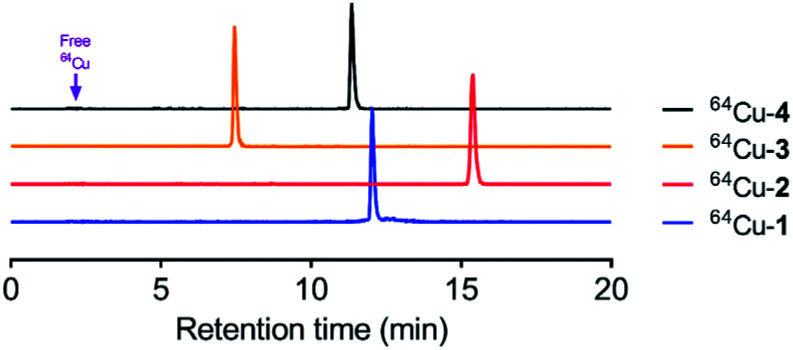
HPLC chromatograms of ^64^Cu radiolabeled compounds. Retention times were 12.02, 15.4, 7.5 and 11.3 min for ^64^Cu-1, ^64^Cu-2, ^64^Cu-3 and ^64^Cu-4, respectively. If present, free copper would appear at 2.5 min.

Conjugates 1–4 were loaded with ^64^Cu^2+^ (CuCl_2_) to give the corresponding complexes, which were analysed *via* reversed-phase HPLC. Radiochemical purity was determined as 98 ± 1.3%, and specific activity was maintained at *ca.* 20 μCi μg^−1^ for all compounds. As shown in Fig. 2, copper-labelled, ceragenin-NOTA conjugates 1 and 2 (^64^Cu-1 and ^64^Cu-2) gave retention times of 12.0 and 15.4 min, respectively, without loss of the copper label (*i.e.*, free copper was not detected). To the extent that retention times on a C_18_ silica chromatography column provide information about the lipophilicity of analytes, labelled structural variants 3 and 4 (^64^Cu-3 and ^64^Cu-4) proved to have lipophilicities comparable to and less than those of ^64^Cu-1 and ^64^Cu-2.

To further demonstrate the stability of the complexes, studies were conducted in human serum to investigate potential degradation of the radiolabeled compounds. The stability of ceragenin complexes was determined by measuring the radioactive fraction of the parent compound at incubation intervals in human serum at 37 °C. No unbound metal was observed over the evaluation time indicating that the complex remained 100% intact up to 4 h.

To determine the relative uptake and retention of the conjugates, biodistribution experiments were conducted in CD-1 mice with labeled 1–4 (Table S1†). All animal procedures were performed in accordance with the Guidelines for Care and Use of Laboratory Animals of Washington University and experiments were approved by the Animal Ethics Committee of Washington University. The most substantial differences in uptake and retention among the conjugates were that ^64^Cu-1 and ^64^Cu-2 were retained in the liver in higher relative amounts than ^64^Cu-3 and ^64^Cu-4, and that ^64^Cu-1 was retained in the kidney in higher relative amounts than ^64^Cu-2.

**Fig. 3:**
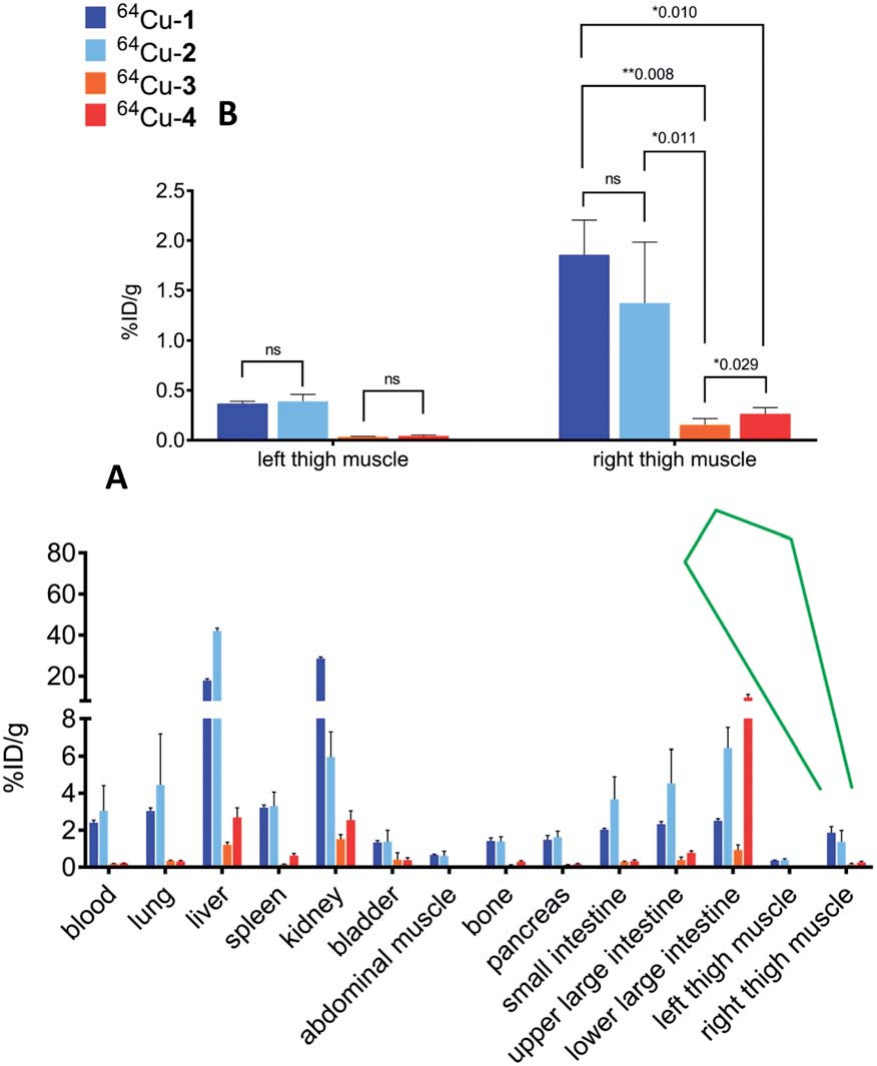
(A) Overall biodistribution results (% ID per g) of ^64^Cu-1, ^64^Cu-2, ^64^Cu-3 and ^64^Cu-4 in CD-1 mice with intramuscular infection in right thigh muscle at 4 h post injection. (B) Expansion of plot of data from left and right thigh muscles.

We used a model of thigh muscle infection with *Escherichia coli* (TG1) in CD-1 mice to evaluate the impact of infection on distribution of the conjugates. The infection was established 24 h before injection of the conjugates. The primary measure of uptake was percentage of injected dose per gram in each type of tissue (% ID per g). We previously observed high affinity of ceragenins for the outer membranes of Gram-negative bacteria, including the lipid A portion of lipopolysaccharide,^6,7^ and it was anticipated that this affinity would result in accumulation of the conjugates at the site of infection. Similar to the uninfected mice (Table S1†), ^64^Cu-1 and ^64^Cu-2 accumulated in the liver and kidney, relative to ^64^Cu-3 and ^64^Cu-4 (Fig. 3) of infected mice. Uptake values for labelled conjugates in the left thigh muscle (control, no bacteria) were not significantly different (Fig. 3B). Uptake in the right thigh muscle, containing the bacterial infection, was highest with ^64^Cu-1 (1.86 ± 0.78% ID perg) and was lowest with ^64^Cu-3 (0.26 ± 0.07% ID per g) with a p value of 0.008. The uptake of ^64^Cu-2 was also significantly increased relative to ^64^Cu-3 and ^64^Cu-4.

**Fig. 4:**
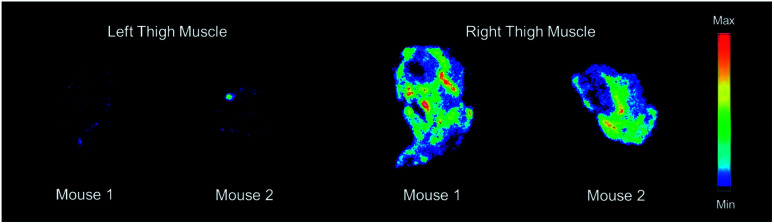
Representative images from *ex vivo* autoradiography of left and right thigh muscle sections (20–40 μm) from ^64^Cu-1 in CD-1 mice, at 4 h post injection.

**Fig. 5:**
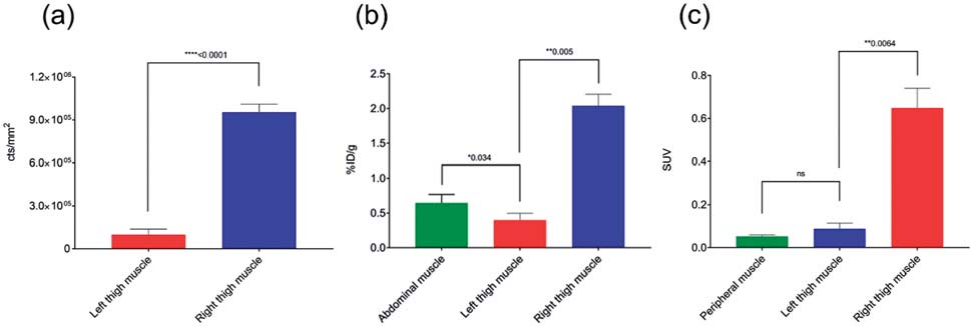
Comparisons of ^64^Cu-1 uptake in CD-1 mice with an intramuscular infection (*E. coli*) in the right thigh at 4 h post injection. (a) Quantitative results (counts per mm^2^) of ^64^Cu-1 in an *ex vivo* autoradiography study with left and right thigh muscle sections (20–40 μm, *n* = 9 sections per muscle). (b) Abdominal *vs.* thigh muscle uptake of ^64^Cu-1 in % ID per g. (c) SUV (standardized uptake value) calculations from *in vivo* imaging comparing peripheral and thigh muscle.

Based on the biodistribution results, ^64^Cu-1 was selected for more detailed autoradiography studies of infections sites. Thigh infections (*E. coli*) were generated in mice as described above, and muscles were removed, sectioned, and imaged. Uptake of ^64^Cu-1 was much higher in the infected thigh muscles compared to the control left thigh (Fig. 4). This significant difference (*p* < 0.0001) was quantified by recording the number of counts per mm^2^ from the sections and these values are given in Fig. 5A. A comparison of the % ID per g of ^64^Cu-1 in abdominal and thigh muscles is given in Fig. 5B. Further comparison was from *in vivo* measurement of standardized uptake values (SUVs), which are ratios of the image-derived radioactivity concentration and the whole-body concentration of injected radioactivity. With this measurement, there is no significant difference between the uptake of ^64^Cu-1 in uninfected thigh muscle and peripheral muscle, while the difference between uptake in infected and uninfected muscle is significant (*p* = 0.0064) (Fig. 5C).

**Fig. 6:**
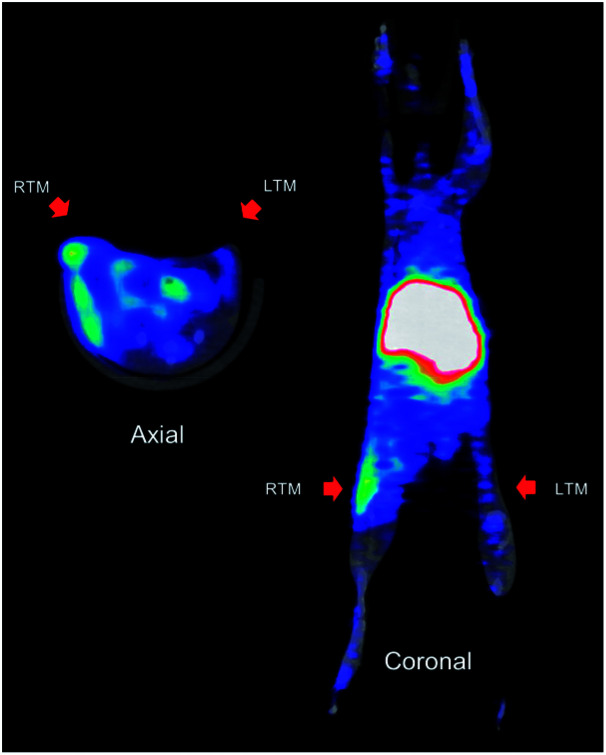
Representative coronal and axial PET images of ^64^Cu-1 in athymic nude mice, with intramuscular infection in right thigh muscle, at 4 h post injection. Left and right thigh muscles indicated in the figure in red arrows.

In biodistribution studies of ^64^Cu-1 (Fig. 3), we saw accumulation of the conjugate in the liver and kidneys, and with *in vivo* imaging we saw similar accumulation. A representative image of PET static data in CD-1 mice with infection in the right thigh is shown in Fig. 6. Along with a kidney and liver uptake, the PET image gave a clear indication of specific infection labelling of the right thigh compared to the left.

## Conclusions

Lipopolysaccharide is the primary constituent of the outer membranes of Gram-negative bacteria, and the lipid A structure is well conserved among bacteria. Consequently, lipid A is a logical target for bacterial recognition. We have measured the *K*_d_ of the base structure of ceragenins for the lipid A portion of lipopolysaccharide as 0.59 μM,^7^ and the affinity of this structure for lipid A is greater than that of polymyxin B, the paradigm of small molecule binding of lipid A. The affinity of ceragenins for lipid A is consistent with measurements of interactions of the ceragenin base structure for intact cells; a labelled ceragenin was shown to preferentially associate with Gram-negative bacteria over Gram-positive bacteria and eukaryotic cells.^7^ Translation of affinity of ceragenins to imaging of infection poses a challenge due to the complex environments provided by *in vivo* applications including higher numbers of eukaryotic cells relative to prokaryotes. Nevertheless, by conjugating ceragenins to NOTA and loading with ^64^Cu, we have generated compounds that selectively label infected tissue in mice. These conjugates display the expected ability to bind ^64^Cu while retaining affinity for bacteria. This affinity allows selective labelling of bacteria and may provide a means of identifying sites of infection in patients that display associated symptoms without indications of localized infection.

## Conflicts of interest

PBS is a paid consultant for CSA Biotechnologies, Inc. Other authors declare no competing financial interest.

## Supplementary Material

RA-009-C9RA02226K-s001
