# Supplementary material for: Translation of ceragenin affinity for bacteria to an imaging reagent for infection
Source: RSC Adv. 2019 May 8;9(25):14472–6. doi: 10.1039/c9ra02226k (PMC7451630; doi:10.1039/c9ra02226k)
Supplement: RA-009-C9RA02226K-s001 [file RA-009-C9RA02226K-s001.pdf]

This journal is © The Royal Society of Chemistry 2019

Supplementary Information

^{1}H NMR and ^{13}C NMR spectra were recorded on Varian Unity 500 MHz instrument. Mass spectrometric data were obtained on Agilent 1100 series spectrometer. All solvents used in the experiments were dried by passage through a Glass Contour solvent drying system containing cylinders of activated alumina. Chemicals were obtained from Sigma-Aldrich, Acros, TCI or Fluka and were used as received unless otherwise noted. All solutions and buffers were prepared using water purified from a Millipore Integral 5 Milli-Q water system (18 MΩ·cm resistivity, Billerica, MA). The buffer was then treated with Chelex overnight and filtered through a 0.22 μm nylon filter to remove trace amounts of metal ions. Radioactivity was counted with a Beckman Gamma 8000 counter containing a NaI crystal (Beckman Instruments, Inc., Irvine, CA). High-performance liquid chromatography (HPLC) analysis was performed using Kinetex (Phenomenex) reverse phase C-18 column (5 μm, 4.6 x 150 mm I.D.) in Agilent Technologies 1200 series HPLC equipped with a NaI radiotracer detector and a photodiode array detector.

Radiolabeling

^{64}Cu ($t_{1/2}$= 12.7 h, β^{+}=17%, β^{-}=39%, EC=43%, E_{max}=0.656 MeV) was produced by a (p,n) reaction on enriched ^{64}Ni on a TR-19 biomedical cyclotron (Advanced Cyclotron Systems Inc., Richmond, B.C., Canada) at Department of Radiology, Washington University School of Medicine and purified with an automated system using standard procedures.[S1,S2] A stock solution of $^{64}CuCl_2$ was diluted with a 10-fold excess of 0.1 *M* ammonium acetate (NH_4OAc), pH 5.5. Typical labeling of compounds was achieved by adding 10 μg of conjugates to 37 MBq (1 mCi) of $^{64}CuCl_2$ in 100 μL of 0.1 *M* NH_4OAc (pH 5.5). The reactions were incubated on a thermomixer with 800 rpm agitation at 37 ºC for 1 h. Radiolabeled compounds were analyzed by high-performance HPLC, with water (0.1% TFA) and acetonitrile (0.1% TFA) mobile phase with a gradient of 0-100% acetonitrile over 20 min with a 1 mL / min flow rate. A radiochemical yield of greater than 98% was achieved for all labeled compounds and therefore used without further purification.

Serum Stability Studies

Aliquots of 10 μL (~100 μCi) of ^{64}Cu labeled CSA complexes were added to 90 μL of human serum (Sigma-Aldrich) and incubated at 37 °C with agitation (500 rpm). Aliquots were removed at each time point and analyzed using HPLC with the use of an additional guard column, (Phenomenex SecurityGuard 3.00 mm I.D.) for the protection of the C-18 phenomenex column. Samples were evaluated at 0.5, 1, 2, and 4 h. All reactions were conducted in triplicate.

***In vitro* Binding Assay with ^{64}Cu-Radiolabeled Complexes**

Escherichia coli TG1 cells were grown in SOC media (2% w/v tryptone, 0.5% w/v yeast extract, 8.56 mM, 2.5 mM NaCl, 2.5 mM KCl, 10 mM $MgCl_2$ and 20 mM glucose) overnight at 37 ^{o}C with 215 rpm shaking. The culture was inoculated from OD620 0.1 to 1. The growth medium was washed with PBS, and after 1000 rpm centrifugation, cells were resuspend in PBS to

minimize lysis. The ^{64}Cu radiolabeled **CSA-150** and **Control-150** complexes were added at varied concentrations to populations of bacteria from 10^6 to 10^{10} in PBS (100 µL). The mixtures were incubated at 25°C for 1 h, samples were centrifuged, supernatants were aspirated, and the cells were washed twice with cold PBS. The amount of bacteria-associated radioactivity was determined by gamma counting on a Packard II gamma counter (Perkin Elmer, Boston, MA), and data were plotted in GraphPad Prism 7 Software.

Biodistribution Studies

All animal experiments were performed in compliance with the Guidelines for Care and Use of Research Animals established by the Division of Comparative Medicine and the Animal Studies Committee of Washington University School of Medicine. Initial biodistribution studies were conducted in female, 4-5 week-old, wild type CD-1 mice (n = 4, Charles River Laboratories) weighing 16.8 ± 0.7 g. The injection dose was prepared by diluting the radiolabeled activity into 90% saline. Animals were injected with 0.30-0.37 MBq (6 - 10 µCi) of ^{64}Cu-CSA complexes via the tail vein while anesthetized with 2% isoflurane. After each time point (30 min, 2, and 4 h), mice were anesthetized and euthanized by cervical dislocation. Blood, liver, kidney, and other organs of interest were harvested. For infection studies, female, 5-6 week-old, CD-1 mice (Charles River Laboratories) were used (n = 5) weighing 19.5 ± 1.9 g. An intramuscular infection (IM) was established in the right thigh muscle by injecting approximately 5 x10^9 bacteria cells suspended in PBS (100 µL). The IM infection was allowed to progress for 24 h, followed by administration of the radioactive material via the tail vein. Organs were harvested at 4 h post injection and the amount of radioactivity in each organ was determined by gamma counting and percent injected dose per gram of tissue (%ID/g) calculated (Table S1). Quantitative data were processed by Prism 7 (GraphPad Software, v 7.04, San Diego, CA) and expressed as mean ± SD. Statistical analysis performed using one-way analysis of variance and Student's t test. Differences at the 95% confidence level ($p < 0.05$) were considered statistically significant.

Table S1. Biodistribution results of ^{64}Cu-**1,** ^{64}Cu-**2,** ^{64}Cu-**3** and ^{64}Cu-**4** in CD-1 mice with intramuscular infection in right thigh muscle, at 4 h post injection. Data are reported as the percent of the injected dose per gram of tissue (mean ± SD).

	^{64}Cu-**1**	^{64}Cu-**2**	^{64}Cu-**3**	^{64}Cu-**4**
blood	2.41 ± 0.30	3.05 ± 1.37	0.19 ± 0.03	0.20 ± 0.03
lung	3.04 ± 0.39	4.45 ± 2.74	0.35 ± 0.03	0.31 ± 0.06
liver	17.96 ± 1.60	42.01 ± 1.48	1.21 ± 0.14	2.68 ± 0.54
spleen	3.22 ± 0.33	3.31 ± 0.76	0.16 ± 0.02	0.63 ± 0.13
kidney	28.62 ± 1.88	5.95 ± 1.33	1.52 ± 0.25	2.54 ± 0.51
bladder	1.34 ± 0.25	1.39 ± 0.61	0.40 ± 0.38	0.38 ± 0.15
abdominal muscle	0.66 ± 0.12	0.61 ± 0.26	0.04 ± 0.01	0.05 ± 0.01
bone	1.41 ± 0.41	1.39 ± 0.27	0.10 ± 0.03	0.29 ± 0.06
pancreas	1.48 ± 0.55	1.63 ± 0.32	0.13 ± 0.01	0.17 ± 0.04
small intestine	2.02 ± 0.20	3.68 ± 1.22	0.28 ± 0.05	0.33 ± 0.07
upper large intestine	2.32 ± 0.34	4.53 ± 1.84	0.39 ± 0.16	0.77 ± 0.12
lower large intestine	2.50 ± 0.28	6.42 ± 1.11	0.93 ± 0.28	9.64 ± 1.59
left thigh muscle	0.37 ± 0.05	0.39 ± 0.07	0.03 ± 0.01	0.04 ± 0.01
right thigh muscle	1.86 ± 0.78	1.37 ± 0.61	0.15 ± 0.06	0.26 ± 0.07

Ex vivo Autoradiography

From the initial biodistribution studies with wild type CD-1 mice, freshly harvested kidneys were immediately frozen by immersion in liquid nitrogen and sectioning was carried out using a whole body cryo-microtome (Vibratome 8850, SIMS Co., Ltd, Tokyo, Japan). For the IM infection study, abdominal, and left and right thigh muscles were harvested from the CD-1 mice and immersed in 4% formaldehyde (diluted from 32% formaldehyde, Sigma-Aldrich) overnight, and sectioned using a cryostat (CM1860, Leica Biosystems Inc., Wetzlar, Germany). Each organ was cut into 20-40 μm sections and attached to adhesive glass slides (CFSA 1X, Leica Biosystems Inc.). Sections were exposed to a phosphor imaging plate (GE Healthcare Life Sciences) for 12 - 14 h and the plates were scanned using phosphor imager plate scanner (Storm 840). The resulting images were processed using ImageQuant 5.2 (Molecular Dynamics) and ImageJ (v1.48, public domain) software.

PET/CT Imaging Studies

Small animal PET/CT imaging study was conducted in 5-7 weeks old female CD-1 mice (n = 4, 20.2 ± 0.9 g) with intramuscular infection in the right thigh muscle. Approximately 3.70 MBq (100 μCi) of ^{64}Cu-**CSA-152** was administrated via tail vein injection. Mice were anesthetized with 1-2% isofluorane/oxygen mixture and imaged on an Inveon small animal PET/CT scanner (Siemens Medical Solutions) at 4 h after administration. Static images were collected for 15 min and reconstructed with the Maximum Aposteriory Probability (MAP) algorithm followed by CT co-registration with the Inveon Research Workstation image display software (Siemens Medical Solutions, Knoxville, TN).

Post-PET Biodistribution

After *in vivo* imaging at 4 h, the mice were euthanized and subjected to biodistribution studies.

Preparation of 7a: To a solution of **5a** (4.67 g, 3.8 mmol) in acetonitrile (100 mL), potassium carbonate (1.5 g, 3.8 mmol) and **6** (1.45 g, 3.8 mmol) were added sequentially. The mixture was heated to reflux for 12 h. The mixture was allowed to cool to room temperature and water (50 mL) was added. The product was extracted with ethyl acetate (100 mL), and the extract was dried over sodium sulfate and concentrated under vacuum. Column chromatography (silica gel with dichloromethane/methanol: 20:1 as eluent) gave the desired azide as 2.8 g (64% yield) of a clear oil. ^{1}H NMR ($CDCl_3$, 500 MHz): δ 4.79 (br, 1 H), 3.6-3.7 (m, 18 H), 3.03-3.58 (m, 22 H), 1.55-2.11 (m, 17 H), 1.43 (s, 27 H), 1.20-1.31 (m, 9 H), 0.98-1.03 (m, 2 H), 0.88-0.94 (m, 8 H), 0.69 (s, 3 H). ^{13}C NMR ($CDCl_3$, 125 MHz): δ 158.02, 156.23,156.07, 80.56, 79.26, 78.89, 78.63, 70.70, 70.67, 70.63, 70.61, 70.57,70.52, 70.15, 70.02, 66.40, 65.76, 50.61, 47.78, 47.05, 46.15, 42.60, 41.88, 41.84, 39.68, 39.43,38.64, 38.35, 35.66, 35.21, 34.91, 34.86, 33.16, 31.83, 30.57, 30.11, 29.69, 29.47, 29.27, 28.72, 28.61, 28.54, 28.48, 17.89, 14.11, 12.49. HRMS (ESI) calcd for $C_{59}H_{108}N_8O_{14}$ $[M+H]^+$: 1153.7985, found: 1153.8031.

Preparation of 7**b:** Phenylcarbonate **6** (30 mg, 0.075 mmol) was added to a mixture of tris-Boc CSA-13 (**5b**) (73 mg, 0.075 mmol) and potassium carbonate (21 mg, 0.15 mmol) in acetonitrile (3 mL). The mixture was refluxed for 12 h. Water (5 mL) was added after the reaction mixture had been cooled to room temperature. The resulting mixture was extracted with ethyl acetate (3 x 5 mL), and the combined organic extracts were dried over sodium sulfate. The solvent was removed under vacuum, and the product was purified via silica gel chromatography (5% methanol in dichloromethane as eluent). Compound **7b** was recovered as a clear oil (63 mg, 67% yield). ^{1}H NMR ($CDCl_3$, 500 MHz): δ 4.79 (br, 1 H), 3.6-3.7 (m, 18 H), 3.03-3.58 (m, 24 H), 1.55-2.11 (m, 17 H), 1.43 (s, 27 H), 1.20-1.31 (m, 21 H), 0.98-1.03 (m, 2 H), 0.88-0.94 (m, 11 H), 0.69 (s, 3 H). ^{13}C NMR ($CDCl_3$, 125 MHz): δ 158.02, 156.23,156.07, 80.56, 79.26, 78.89, 78.63, 70.70, 70.67, 70.63, 70.61, 70.57,70.52, 70.15, 70.02, 66.40, 65.76, 50.61, 47.78, 47.37, 47.05, 46.15, 42.60, 41.88, 41.84, 39.68, 39.43,38.64, 38.35, 35.66, 35.21, 34.91, 34.86, 33.16, 31.83, 30.57, 30.11, 29.69, 29.47, 29.27, 28.72, 28.61, 28.54, 28.48, 27.61, 27.04, 25.63, 23.31, 22.95, 22.87, 22.48, 17.89, 14.11, 12.49. HRMS (ESI) calcd for $C_{67}H_{124}N_8O_{14}$ $[M+H]^+$: 1265.9237, found:1265.9648.

Preparation of 1: To a solution of **7a** (2.8 g, 2.4 mmol) in THF (50 mL) and water (17 mL), triphenyl phosphine (6.2 g, 24 mmol) was added. The mixture was stirred at room temperature for 12 h. The THF was removed under vacuum, and the resulting mixture was exacted with dichloromethane (3 x 50 mL). The combined extracts were dried over sodium sulfate and concentrated under vacuum. A silica gel plug, washed with dichloromethane/methanol: 20:1, was used to remove triphenylphosphine oxide and excess triphenylphosphine giving the desired amine in crude form as 1.8 g (73% yield) of a clear oil. To a solution of the amine (0.3 g, 0.27 mmol) and bis-BOC NOTA (0.11 g, 0.28 mmol) in DMF (20 mL), COMU (0.14 g, 0.33 mmol)

and collidine (0.05 g, 0.416 mmol) were added. The mixture was stirred for 2 h. Water (20 mL) was added, and the mixture was extracted with dichloromethane (3 x 50 mL). The combined extracts were dried over sodium sulfate and concentrated under vacuum. Column chromatography (silica gel with dichloromethane/methanol 20:1 as eluent) gave the desired conjugate as 0.25 g (65% yield) of a clear oil. ^{1}H NMR ($CDCl_3$, 500 MHz): δ 4.79 (br, 1 H), 3.6-3.7 (m, 18 H), 3.03-3.58 (m, 28 H), 22-2.4 (m,12H), 1.55-2.11 (m, 17 H), 1.43 (s, 27 H), 1.39 (s, 18H), 1.20-1.31 (m, 9 H), 0.98-1.03 (m, 2 H), 0.88-0.94 (m, 8H), 0.69 (s, 3 H). ^{13}C NMR ($CDCl_3$, 125 MHz): δ 158.02, 156.23,156.07, 82.3, 81.18, 80.56, 79.26, 78.89, 78.63, 70.70, 70.67, 70.63, 70.61, 70.57,70.52, 70.15, 70.02, 69.78, 69.66, 69.59, 69.51, 69.50, 69.45, 66.48, 66.47, 66.45, 66.40, 65.76, 50.61, 47.78, 47.05, 46.15, 42.60, 41.88, 41.84, 39.68, 39.43,38.64, 38.35, 35.66, 35.21, 34.91, 34.86, 33.16, 31.83, 30.57, 30.11, 29.69, 29.47, 29.27, 28.78, 28.72, 28.61, 28.54, 28.48, 25.56, 17.89, 14.11, 12.49. HRMS (ESI) calcd for $C_{79}H_{145}N_9O_{19}$ $[M+H]^+$: 1525.0657, found:1525.0721. The conjugate (0.25 g) was dissolved in a solution of HCl in dioxane (4 *M*, 10 mL) and stirred at room temperature for 18 h. The solvent was removed under vacuum, and toluene (50 mL) as added. Removal of the toluene under vacuum (azeotropically drying the product) gave **1** (0.15 g, 91% yield) as a light yellow solid. ^{1}H NMR ($CDCl_3$, 500 MHz): δ 4.79 (br, 1 H), 3.6-3.7 (m, 18 H), 3.03-3.58 (m, 28 H), 22-2.4 (m, 12H), 1.55-2.11 (m, 17 H), 1.20-1.31 (m, 9 H), 0.98-1.03 (m, 2 H), 0.88-0.94 (m, 8H), 0.69 (s, 3H). ^{13}C NMR ($CDCl_3$, 125 MHz): δ 170.72, 158.02, 156.07, 80.56, 79.26, 78.89, 70.70, 70.67, 70.63, 70.61, 70.57,70.52, 70.15, 70.02, 69.78, 69.66, 69.59, 69.51, 69.50, 69.45, 66.48, 66.47, 66.45, 66.40, 65.76, 50.61, 47.78, 47.05, 46.15, 42.60, 41.88, 41.84, 39.68, 39.43, 38.64, 38.35, 35.66, 35.21, 34.91, 34.86, 33.16, 31.83, 30.57, 30.11, 29.69, 29.51, 29.47, 29.35, 29.27, 28.78, 28.61, 28.54, 17.89, 14.11, 12.49. HRMS (ESI) calcd for $C_{56}H_{105}N_9O_{13}$ $[M+H]^+$: 1112.7832, found:1112.8031.

Preparation of 2: To a solution of **7b** (20 mg, 0.015 mmol) in THF (2 mL) and water (0.5 mL), triphenyl phosphine (40 mg, 0.15 mmol) was added. The mixture was stirred for 12 h at room temperature. The THF was removed under vacuum, and the resulting mixture was extracted with dichloromethane (3 x 10 mL). The combined organic layers were dried over sodium sulfate and concentrated under vacuum. Column chromatography (silica gel with dichloromethane/methanol 20:1, with 0.05% ammonium hydroxide, as eluent) gave 15 mg (85% yield) of the resulting amine as a clear oil. This amine (15 mg, 0.0125 mmol) and bis-BOC NOTA (5.2 mg, 0.0125 mmol) were dissolved in DMF (1 mL), COMU (6.5 mg, 0.015 mmol) and collidine (2.3 mg, 0.0188 mmol) were added. The mixture was stirred for 2 h. Water (2 mL) was added, and the mixture was exacted with dichloromethane (3 x 5 mL). The combined extracts were dried over sodium sulfate and concentrated under vacuum. Column chromatography (silica gel with dichloromethane/methanol 20:1 as eluent) gave the desired conjugate as 12 mg (63% yield) of a clear oil, which was treated with HCl in dioxane (3 mL, 4 *M*). The mixture was stirred for 18 hours at room temperature. Solvent was removed under vacuum to give **2** (8 mg,91% yield) as a white solid. ^{1}H NMR ($CDCl_3$, 500 MHz): δ 4.79 (br, 1 H), 3.6-3.7 (m, 18 H), 3.03-3.58 (m, 30 H), 22-2.4 (m,12H), 1.52-2.11 (m, 19 H), 1.43 (s, 27 H),

1.39 (s, 18H), 1.20-1.31 (m, 19 H), 0.98-1.03 (m, 2 H), 0.88-0.94 (m, 11H), 0.69 (s, 3 H). ^{13}C NMR ($CDCl_3$, 125 MHz): δ 173.2, 171.4, 158.02, 156.23,156.07, 82.3, 81.18, 80.56, 79.26, 78.89, 78.63, 70.70, 70.67, 70.63, 70.61, 70.57,70.52, 70.15, 70.02, 69.78, 69.66, 69.59, 69.51, 69.50, 69.45, 66.48, 66.47, 66.45, 66.40, 65.76, 50.61, 50.11, 47.78, 47.05, 46.15, 42.60, 41.88, 41.84, 39.68, 39.43,38.64, 38.35, 35.66, 35.21, 34.91, 34.86, 33.16, 31.92, 31.83, 30.57, 30.11, 29.69, 29.47, 29.31, 29.28, 29.27, 28.78, 28.72, 28.61, 28.54, 28.48, 26.61, 25.56, 22.78, 17.89, 14.11, 14.10, 12.49. HRMS (ESI) calcd for $C_{64}H_{121}N_9O_{13}$ $[M+H]^+$: 1225.6980, found:1225.7132.

Preparation of 9a: To a solution of **8**[19] (4.89 g, 10 mmol) in THF (100 mL), were added mesylchloride (1.2 g, 11 mmol) and triethylamine (1.2 g, 12 mmol) at 0 °C. The mixture was stirred and allowed to warm to room temperature over 30 min. Water (50 mL) was added, and the product was extracted using dichloromethane (3 x 50 mL). The combined extracts were dried over sodium sulfate, and solvents were removed under vacuum. After the solvent was removed, the oil was dissolved in DMSO (40 mL) and sodium azide (1.3 g, 20 mmol) was added. The resulting mixture was stirred at 80 °C for 12 h. The mixture was cooled to room temperature and water (50 mL) and dichloromethane (100 mL) were added. The solvents were separated, and the aqueous solution was extracted with dichloromethane (2 x 50 mL). Combined extracts were dried over sodium sulfate and concentrated under vacuum. The resulting azide was dissolved in THF (40 mL) and water (10 mL), and triphenylphosphine (5.2 g, 20 mmol) was added. The resulting mixture was stirred for 12 h at room temperature. The THF was removed under vacuum, and the product was extracted from the mixture with dichloromethane (3 x 50 mL). Combined extracts were dried over Na_2SO_4. Column chromatography (silica gel, 3% MeOH in dichloromethane with 0.1% ammonium hydroxide) gave **9a** (3.2 g, 68% yield) as a clear oil. ^{1}H NMR ($CDCl_3$, 500 MHz) δ 3.16-3.31 (m, 6H), 3.06 (m, 2 H), 2.58 (m, 4 H), 1.64 (m, 4 H), 1.41 (s, 19 H), 1.25-1.3 (m, 2 H), 1.0-1.12 (m, 7 H), 0.68 (t, 3 H). ^{13}C NMR ($CDCl_3$, 125 MHz) δ 156.81, 156.52, 79.81, 79.65, 79.58, 28.4, 27.16, 25.93, 24.41, 23.64, 22.65. HRMS (ESI) calcd for $C_{24}H_{48}N_4O_6$ $[M+H]^+$:489.3574, found:489.4132.

Preparation of 9b: To a solution of **8**[19] (4.89 g, 10 mmol) in THF (100 mL), were added mesylchloride (1.2 g, 11 mmol) and triethylamine (1.2 g, 12 mmol) at 0 °C. The mixture was allowed to warm to room temperature over 30 min. Water (50 mL) was added, and the product was extracted using dichloromethane (3 x 50 mL). The combined extracts were dried over sodium sulfate, and solvents were removed under vacuum. The resulting oil was dissolved in octyl amine (10 mL), and the resulting mixture was stirred at 80 °C for 1 h. The mixture was allowed to cool to room temperature, and water (50 mL) and dichloromethane (100 mL) were added. The layers were separated and the aqueous layer was extracted with dichloromethane (3 x 50 mL). The combined extracts were dried over sodium sulfate and concentrated under vacuum. After chromatography (silica gel, 3% MeOH in dichloromethane with 0.1% ammonium hydroxide), 4.6 g (78% yield) of the desired amine was isolated as a clear oil. ^{1}H NMR ($CDCl_3$, 500 MHz) δ 3.16-3.31 (m, 8 H), 3.06 (m, 2 H), 2.58 (m, 4 H), 1.64 (m, 6 H),

1.41 (s, 27 H), 1.25-1.3 (m, 2 H), 1.0-1.12 (m, 10 H), 0.68 (t, 3 H) ^{13}C NMR ($CDCl_3$, 125 MHz) δ 156.81, 156.52, 79.81, 79.65, 79.58, 28.4, 28.35, 27.16, 25.93, 24.41, 23.64, 22.65, 21.97, 13.42. HRMS (ESI) calcd for $C_{32}H_{64}N_4O_6$ $[M+H]^+$:848.6106, found:848.6183.

Preparation of 10a: To a solution of **9a** (3.2 g, 6.5 mmol) in acetonitrile (30 mL) were added potassium carbonate (1.0 g, 7.6 mmol) and **6** (2.5 g, 6.5 mmol). The mixture was heated to reflux and stirred for 12 h. The mixture was allowed to cool to room temperature, and water (30 mL) was added. The product was extracted with ethylacetate (50 mL), and the resulting solution was dried over sodium sulfate. After chromatography (silica gel, 3% MeOH in dichloromethane) 2.7 g of **10a** (55% yield) was isolated as a clear oil. ^{1}H NMR ($CDCl_3$, 500 MHz) δ 3.6-3.7 (m, 16 H), 3.47 (t, 2 H), 3.36 (t, 2 H),3.16-3.31 (m, 6H), 3.06 (m, 2H), 2.58 (m, 4H), 1.64 (m, 6H), 1.41 (s, 15H), 1.25-1.3 (m, 2H), 1.0-1.12 (m, 7H), 0.68 (t, 3H). ^{13}C NMR ($CDCl_3$, 125 MHz) δ 156.81, 156.52, 154.88, 79.81, 79.65, 79.58, 70.70, 70.67, 70.62, 70.61, 70.57, 70.31, 70.02, 69.85, 41.04, 28.35, 27.16, 24.41, 23.64, 22.65, 21.97. HRMS (ESI) calcd for $C_{35}H_{68}N_8O_{11}$ $[M+H]^+$:777.5008, found:777.5121.

Preparation of 10b: To a solution of **9b** (4.6 g, 7.6 mmol) in acetonitrile (20 mL) were added potassium carbonate (3.0 g, 7.6 mmol) and **6** (2.9 g, 7.6 mmol). The mixture was heated to reflux and stirred for 12 h. The mixture was allowed to cool to room temperature, and water (30 mL) was added. The product was extracted with ethylacetate (50 mL), and the resulting solution was dried over sodium sulfate. After chromatography (silica gel, 3% MeOH in dichloromethane) 3.8 g of **10b** (56% yield) was isolated as a clear oil. ^{1}H NMR ($CDCl_3$, 500 MHz) δ 3.6-3.7 (m, 16 H), 3.47 (t, 2 H), 3.36 (t, 2 H),3.16-3.31 (m, 8 H), 3.06 (m, 2 H), 2.58 (m, 4 H), 1.64 (m, 6 H), 1.41 (s, 27 H), 1.25-1.3 (m, 2 H), 1.0-1.12 (m, 10 H), 0.68 (t, 3 H). ^{13}C NMR ($CDCl_3$, 125 MHz) δ 156.81, 156.52, 154.88, 79.81, 79.65, 79.58, 70.70, 70.67, 70.62, 70.61, 70.57, 70.31, 70.02, 69.85, 50.63, 41.04, 28.4, 28.35, 27.16, 25.93, 24.41, 23.64, 22.65, 21.97, 13.42. HRMS (ESI) calcd for $C_{43}H_{84}N_8O_{11}$ $[M+H]^+$:889.6260, found:889.6254.

Preparation of 3: To a solution of **10a** (2.5 g, 3.2 mmol) in THF (50 mL) and water (10 mL), was added triphenyl phosphine (2.6 g, 10 mmol). The mixture was stirred at room temperature for 12 h. The THF was removed under vacuum, and the remaining aqueous mixture was extracted with dichloromethane (3 x 50 mL). The combined extracts were dried over sodium sulfate, and the desired amine was recovered after chromatography (silica gel, 5% MeOH in dichloromethane with 0.1% ammonium hydroxide) as 1.8 g (77% yield) of a clear oil. This amine (0.5 g, 0.66 mmol) and bis-Boc NOTA (0.27 g, 0.66 mmol), COMU (0.3 g, 0.7 mmol) and collidine (0.1 g, 0.82 mmol) were dissolved in DMF (20 mL). The mixture was stirred for 2 h, and cold water (20 mL) was added. The product was exacted with dichloromethane (3 x 50 mL), and the combined extracts were dried over sodium sulfate. The solvent was removed under vacuum, and after chromatography (silica gel, 3% MeOH in dichloromethane), the desired amide was isolated as a clear oil (0.45 g, 61% yield). ^{1}H NMR ($CDCl_3$, 500 MHz) δ 3.6-3.7 (m, 16 H), 3.47 (t, 2 H), 3.36 (t, 2 H), 3.16-3.31 (m, 12 H), 2.9-3.1 (m,12 H), 3.06 (m, 2 H), 2.58

(m, 4 H), 1.64 (m, 4 H), 1.43 (s, 27 H), 1.41(s, 8 H), 1.25-1.3 (m, 2 H), 1.0-1.12 (m, 7 H), 0.68 (t, 3 H). ^{13}C NMR ($CDCl_3$, 125 MHz) δ 170.71, 168.64, 159.47, 156.81, 156.52, 154.88, 81.18, 80.52, 79.81, 79.65, 79.58, 70.70, 70.67, 70.62, 70.61, 70.57, 70.31, 70.02, 69.85, 66.58, 66.53, 66.50, 66.46, 60.25, 58.06, 56.72, 51.10, 41.04, 28.42, , 27.16, 24.41, 23.64, 22.65, 21.97. HRMS (ESI) calcd for $C_{55}H_{105}N_9O_{16}$ $[M+H]^+$:1149.7679, found:11149.7832. This amide (0.2 g) was dissolved in a solution of HCl in dioxane (4 *M*, 5 mL). The mixture was stirred for 18 h, and solvent was removed under vacuum. Toluene (50 mL) was added to the resulting material and evaporated under vacuum to remove residual water. Compound **3** was isolated as a light yellow solid (0.12 g, 90% yield). ^{1}H NMR (D_2O, 500 MHz) δ 3.96 (m, 4 H), 3.85 (m, 2 H), 3.2-3.6 (m, 34 H), 2.9-3.1 (m, 10 H), 1.98 (m, 2 H), 1.93 (m, 2 H), 0.68 (t, 3 H). ^{13}C NMR (D_2O, 125 MHz) δ 170.71, 168.64, 159.47, 71.51, 70.72, 69.71, 69.54, 69.41, 66.58, 66.53, 66.50, 66.46, 60.25, 58.06, 56.72, 51.10, 51.06, 46.71, 45.06, 44.63, 44.56, 44.30, 43.34, 43.27, 44.14, 39.90, 39.09, 36.49,, 28.35, 27.16, 25.93, 24.41, 23.64, 22.65. HRMS (ESI) calcd for $C_{32}H_{65}N_9O_{10}$ $[M+H]^+$:736.4854, found:736.5023.

Preparation of 4: To a solution of **10b** (3.8 g, 4.3 mmol) in THF (50 mL) and water (17 mL), was added triphenyl phosphine (2.3 g, 8.6 mmol). The mixture was stirred at room temperature for 12 h. The THF was removed under vacuum, and the remaining aqueous mixture was extracted with dichloromethane (3 x 50 mL). The combined extracts were dried over sodium sulfate, and the desired amine was recovered after chromatography (silica gel, 5% MeOH:DCM and 0.1% $NH_4.OH$) as 2.5 g (71% yield) of a clear oil. This amine (0.5 g, 0.58 mmol) and bis-Boc NOTA (0.24 g, 0.58 mmol), COMU (0.3 g, 0.7 mmol) and collidine (0.1 g, 0.82 mmol) were dissolved in DMF (20 mL). The mixture was stirred for 2 h, and water (20 mL) was added. The product was exacted with dichloromethane (3 x 50 mL), and the combined extracts were dried over sodium sulfate. The solvent was removed under vacuum, and after chromatography (silica gel, 3% MeOH in dichloromethane), the desired amide was isolated as 0.40 g (55% yield) of a clear oil. ^{1}H NMR ($CDCl_3$, 500 MHz) δ 3.6-3.7 (m, 16 H), 3.47 (t, 2 H), 3.36 (t, 2 H), 3.16-3.31 (m, 14 H), 2.9-3.1 (m,12 H), 3.06 (m, 2 H), 2.58 (m, 4 H), 1.64 (m, 6 H), 1.43 (s, 27 H), 1.41(s,18 H), 1.25-1.3 (m, 2 H), 1.0-1.12 (m, 10 H), 0.68 (t, 3 H). ^{13}C NMR ($CDCl_3$, 125 MHz) δ 170.71, 168.64, 159.47, 156.81, 156.52, 154.88, 81.18, 80.52, 79.81, 79.65, 79.58, 70.70, 70.67, 70.62, 70.61, 70.57, 70.31, 70.02, 69.85, 66.58, 66.53, 66.50, 66.46, 60.25, 58.06, 56.72, 51.10, 50.63, 41.04, 28.4, 28.35, 27.16, 25.93, 24.41, 23.64, 22.65, 21.97, 13.42. HRMS (ESI) calcd for $C_{63}H_{121}N_9O_{16}$ $[M+H]^+$:1260.8931, found:1260.9032. This amide (0.15 g) was dissolved in a solution of HCl in dioxane (4 *M*, 5 mL). The mixture was stirred for 18 h, and solvent was removed under vacuum. Toluene (50 mL) was added to the resulting material and evaporated under vacuum to remove residual water. Compound **4** was isolated as a light yellow solid (0.09 g, 90% yield). ^{1}H NMR (D_2O, 500 MHz) δ 3.96 (m, 4 H), 3.85 (m, 2 H), 3.2-3.6 (m, 36 H), 2.9-3.1 (m, 10 H), 1.98 (m, 2 H), 1.93 (m, 2 H), 1.0-1.12 (m, 10 H), 0.68 (t, 3 H). ^{13}C NMR (D_2O, 125 MHz) δ 170.71, 168.64, 159.47, 71.51, 70.72, 69.71, 69.54, 69.41, 66.58, 66.53, 66.50, 66.46, 60.25, 58.06, 56.72, 51.10, 51.06, 50.83, 46.71, 45.06, 44.63, 44.56, 44.30, 43.34, 43.27, 44.14, 39.90, 39.09, 36.49, 31.04, 28.57, 28.35, 27.16, 25.93, 24.41, 23.64,

22.65, 21.97, 13.42. HRMS (ESI) calcd for $C_{40}H_{81}N_9O_{10}$ $[M+H]^+$:848.6106, found:848.6183.

Preparation of 6: To a solution of 14-azido-3,6,9,12-tetraoxatetradecan-1-amine[S3] (23.0 g, 88.0 mmol) in dichloromethane (150 mL) and triethylamine (17.0 mL, 125 mmol) was added phenylchloroformate (12.0 mL, 96.5 mmol) dropwise at 0 °C. The resulting mixture was stirred for 1 h, and water (100 mL) was added. The resulting layers were separated, and the aqueous layer was extracted with dichloromethane (3 x 50 mL). The combined dichloromethane extracts were dried over sodium sulfate, and the solvent was removed under vacuum. Compound 3 was purified by passing the material through a silica gel plug (2% methanol in dichloromethane as eluent) yielding a clear oil (32.0 g, 94% yield). ^{1}H NMR ($CDCl_3$, 500 MHz) δ 7.35 (t, 2 H), 7.19 (t, 1 H), 7.12 (d, 2 H), 3.6-3.7 (m, 16 H), 3.47 (t, 2 H), 3.36 (t, 2 H). ^{13}C NMR ($CDCl_3$, 125 MHz) δ 154.88, 151.10 129.24, 125.21, 121.63, 70.70, 70.67, 70.62, 70.61, 70.57, 70.31, 70.02, 69.85, 50.63, 41.04. HRMS (ESI) calcd for $C_{17}H_{26}N_4O_6$ $[M+H]^+$: 383.1822, found: 383.1868.

S1. McCarthy, D.W.; Shefer, R. E.; Klinkowstein, R. E. et al. Efficient production of high specific activity ^{64}Cu using a biomedical cyclotron. *Nucl. Med. Biol.*, **1997** *24*, 35-43.
S2. Kume, M.; Carey, P. C.; Gaehle, G. et al. A semi-automated system for the routine production of copper-64. *Appl. Radiat. Isot.*, **2012**, *70*, 1803-1806.
S3. Schwabacher, A. W.; Lane, J. W.; Schiesher, M. W.; Leigh, K. M.; Johnson, C. W. Desymmetrization reactions: efficient preparation of unsymmetrically substituted linker molecules. *J. Org. Chem.* **1998**, *63*, 1727-1729.

NMR Spectra for 1, 2, 3, 4, 6, 7a, 7b, 9a, 9b, 10a and 10b.

PROTON_Compound 7a

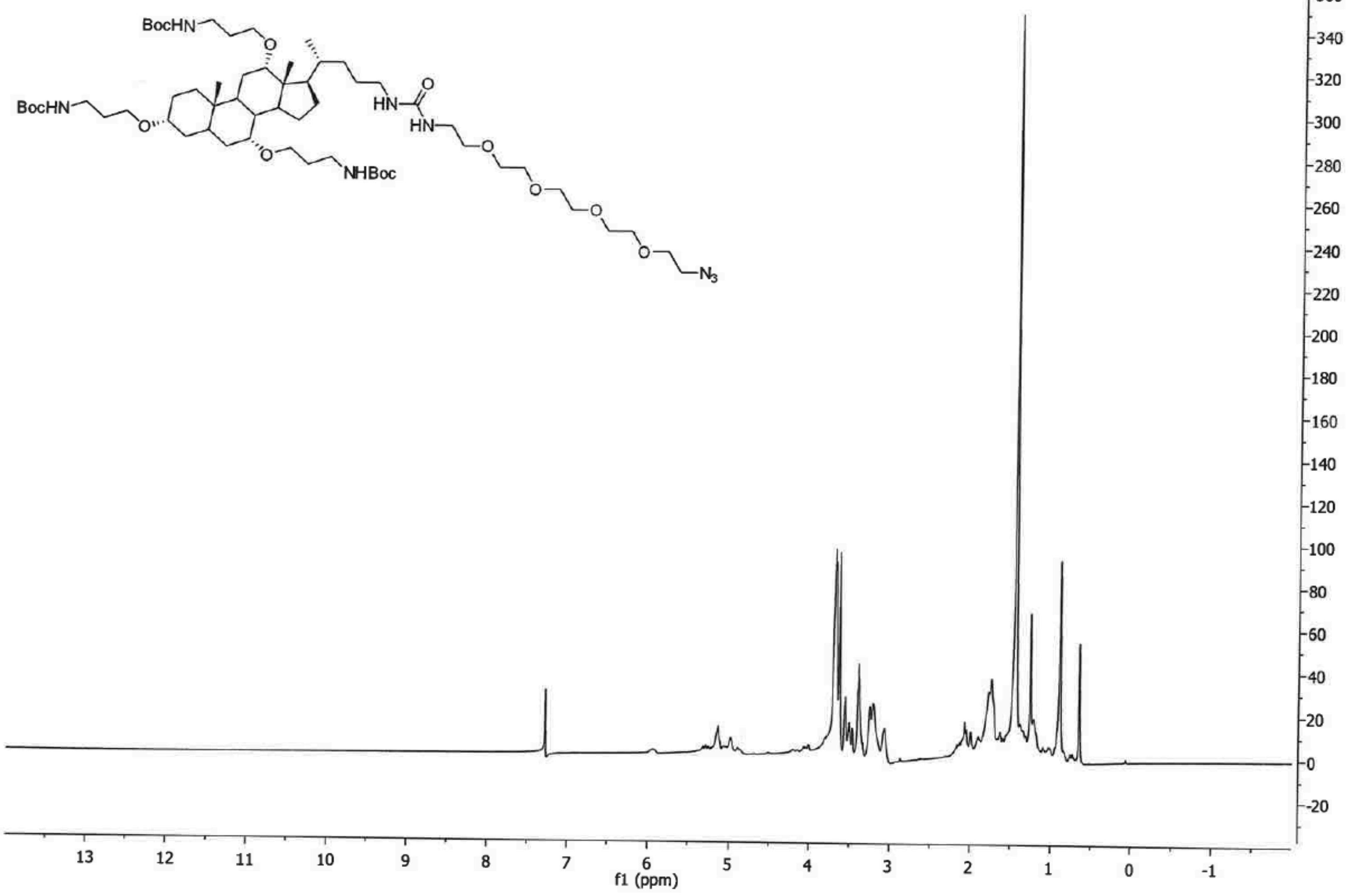

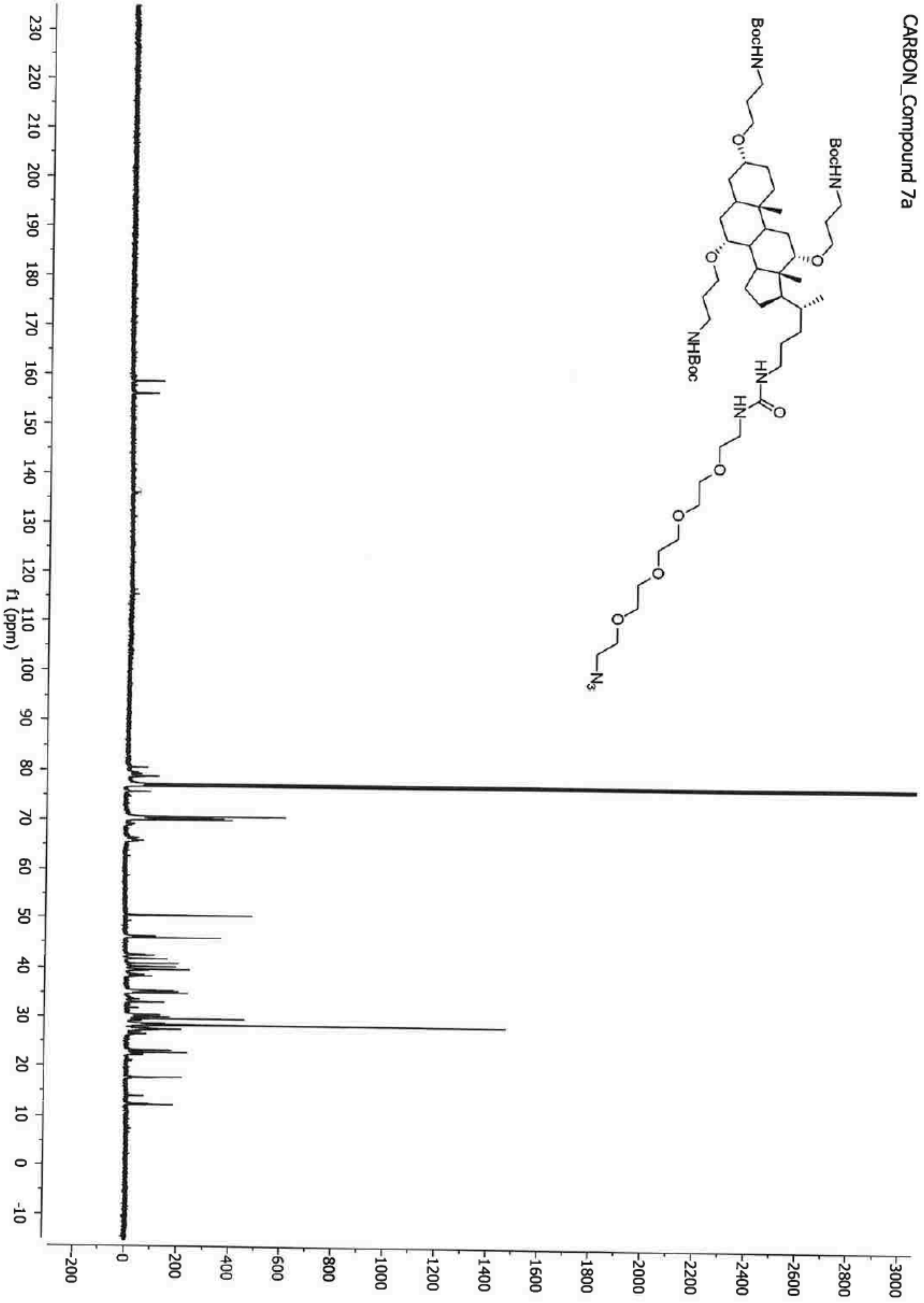
CARBON_Compound 7a
BocHN
BocHN
NHBoc
HN
HN
N_3
f1 (ppm)

PROTON_Compound 7b

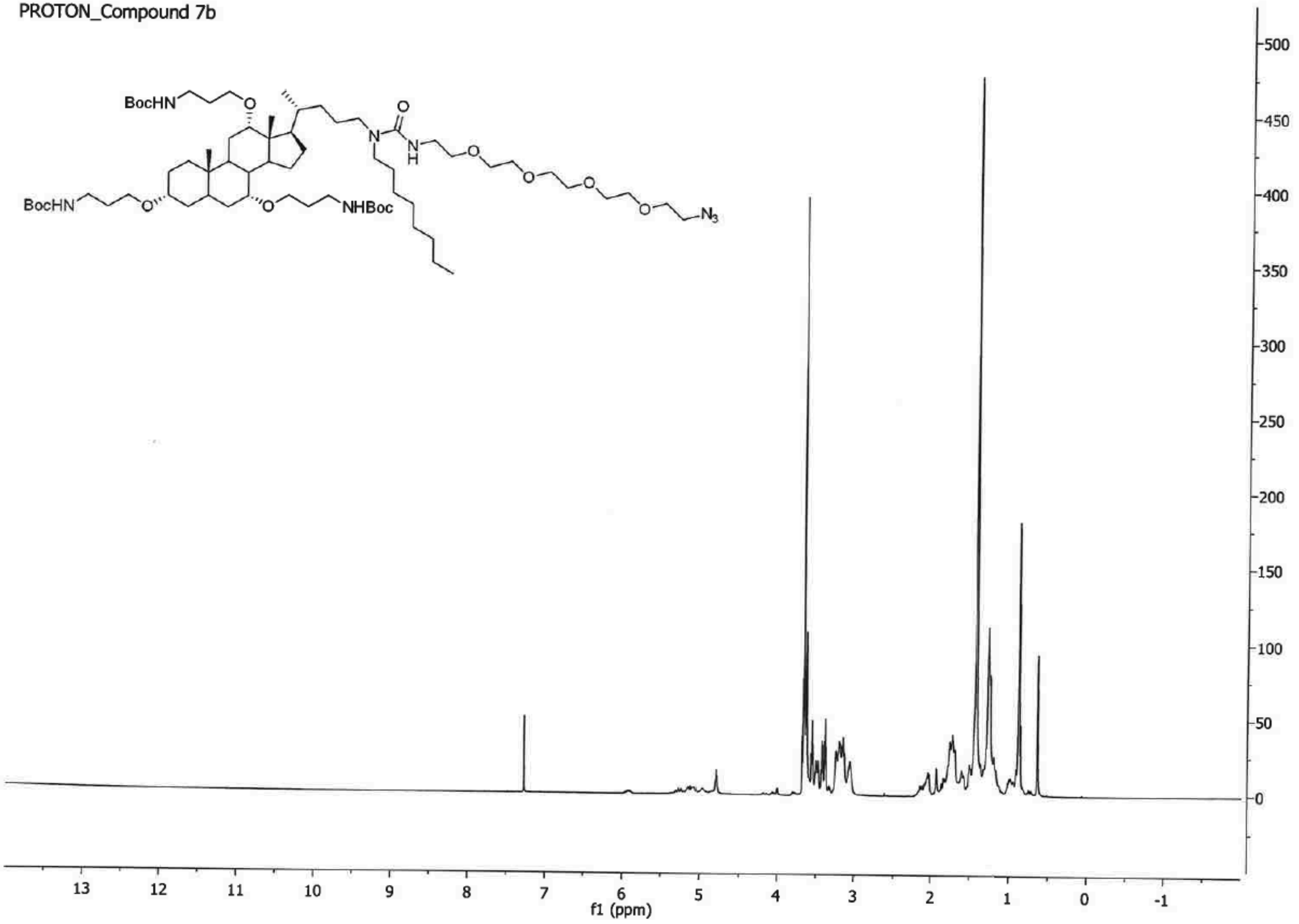

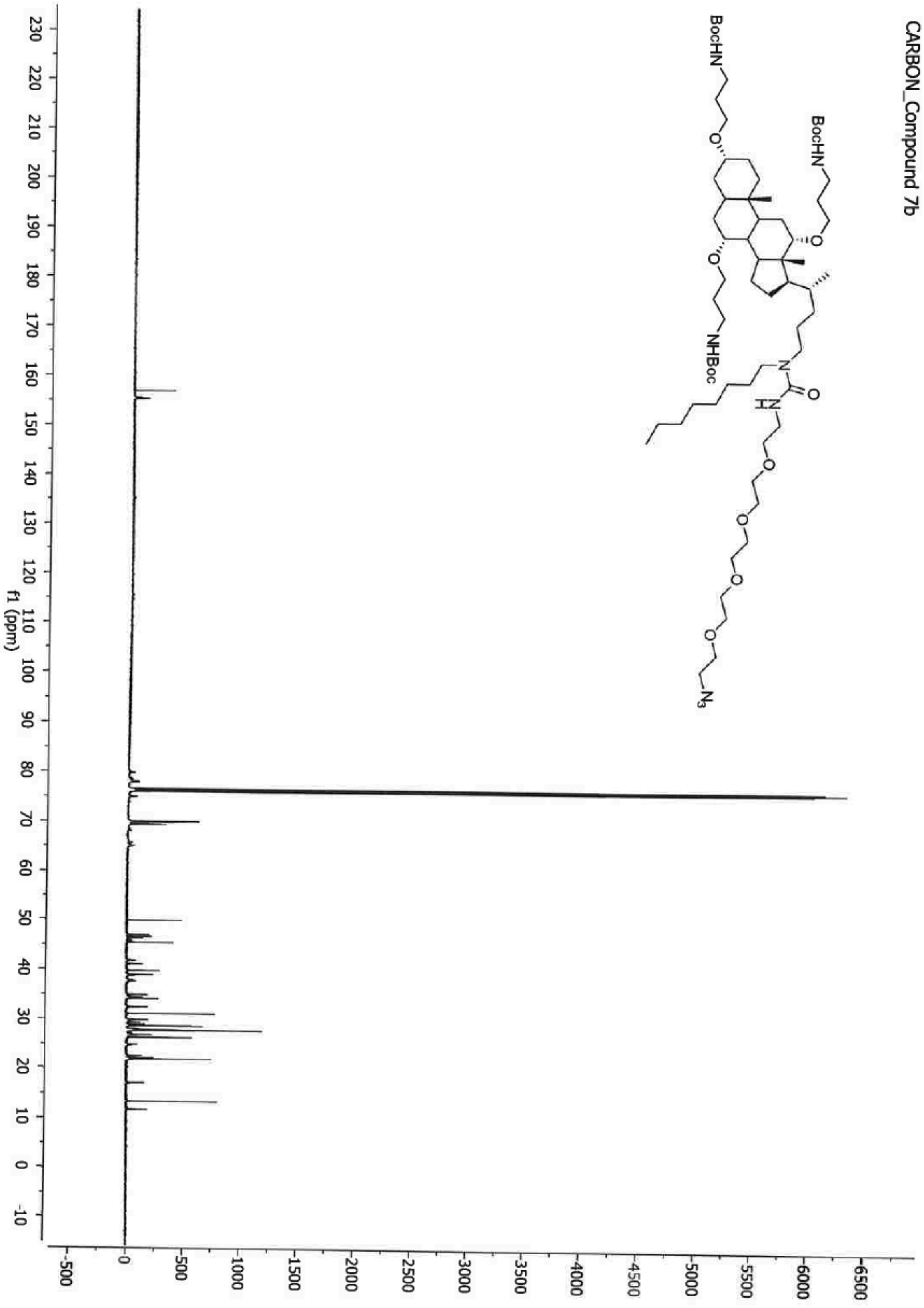

CARBON_Compound 7b
BocHN
BocHN
NHBoc
N
O
H
N
O
O
O
O
O
O
N_3
f1 (ppm)
230 220 210 200 190 180 170 160 150 140 130 120 110 100 90 80 70 60 50 40 30 20 10 0 -10
-500 0 500 1000 1500 2000 2500 3000 3500 4000 4500 5000 5500 6000 6500

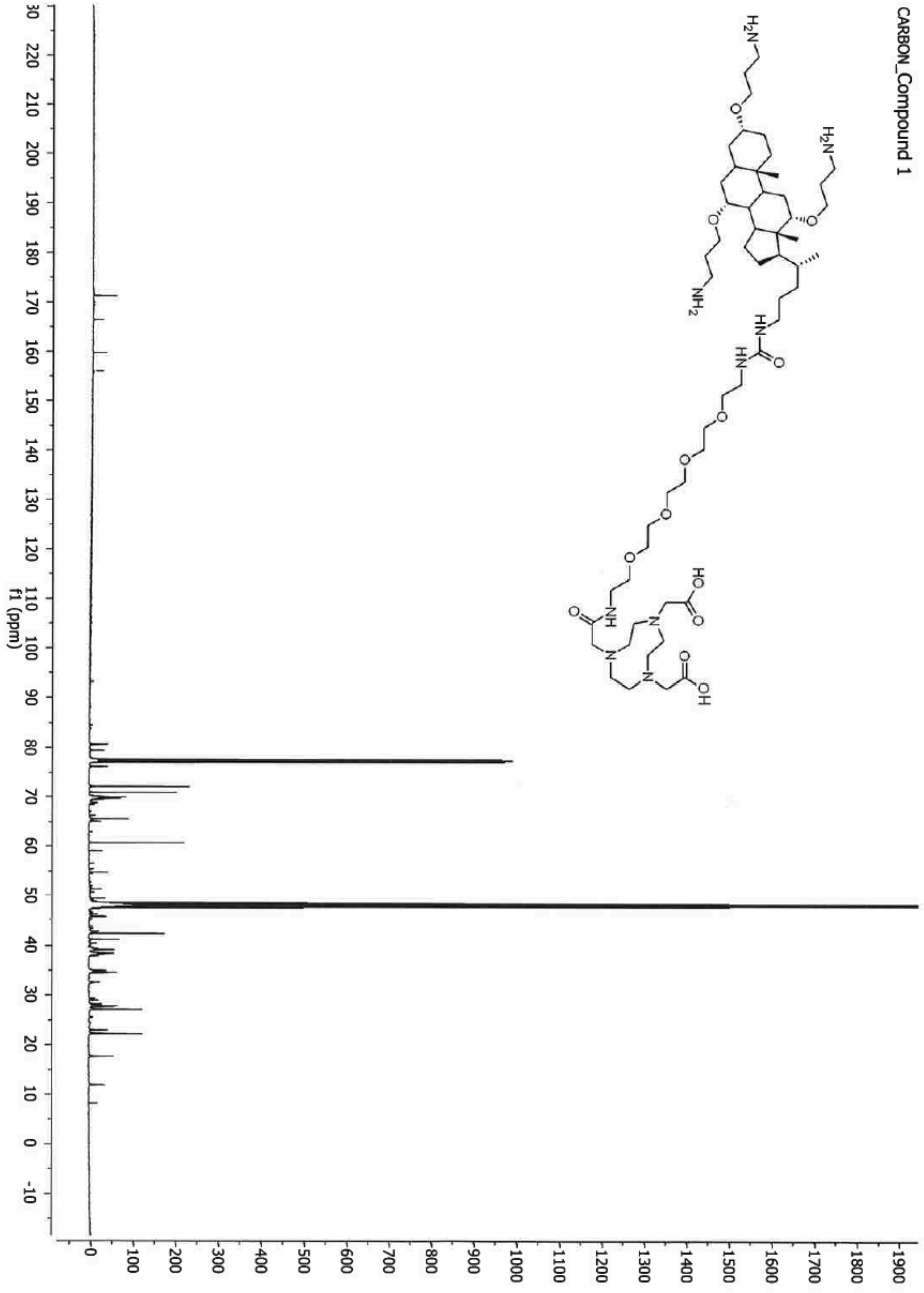

CARBON_Compound 1
f1 (ppm)

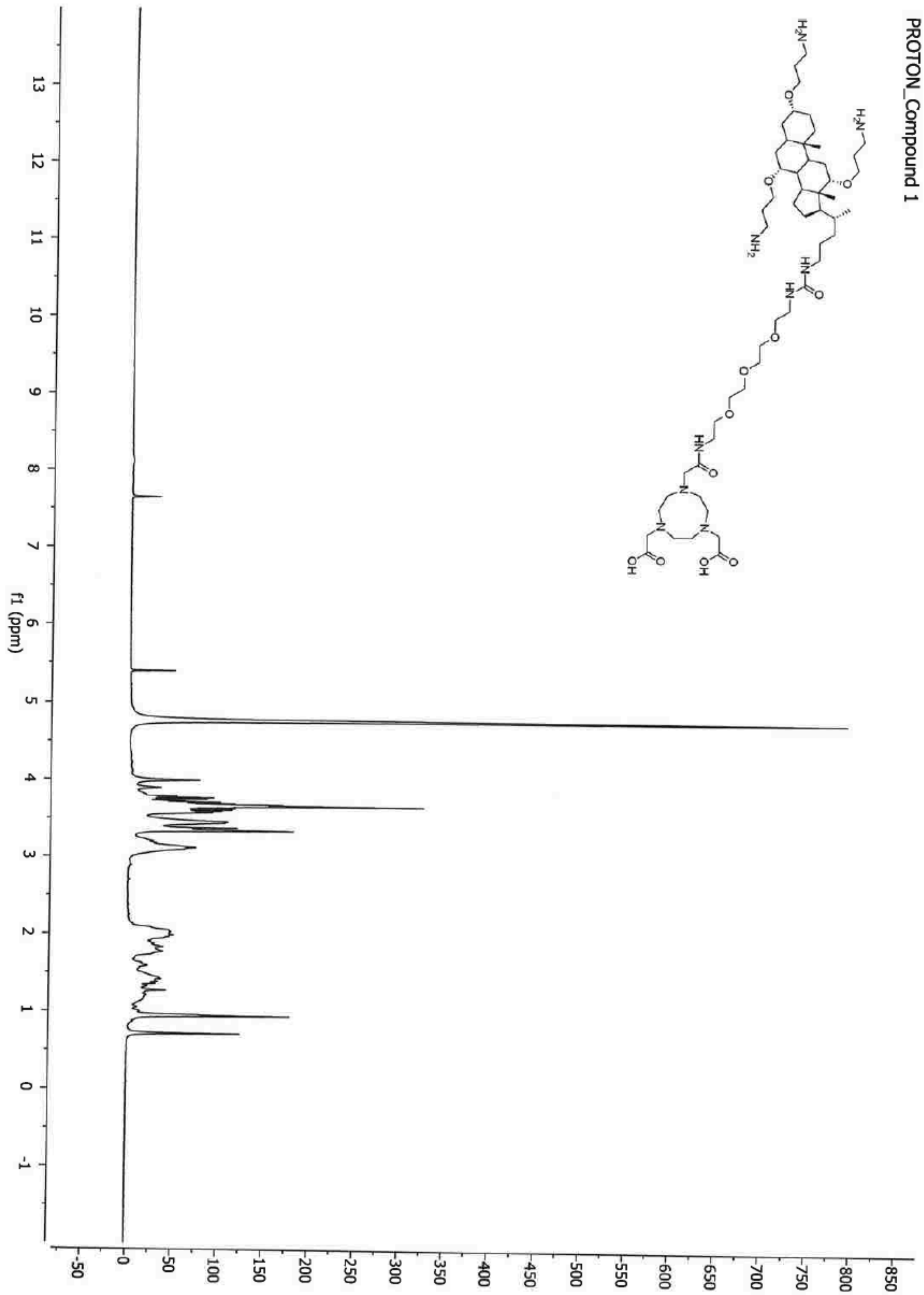
PROTON_Compound 1
f1 (ppm)

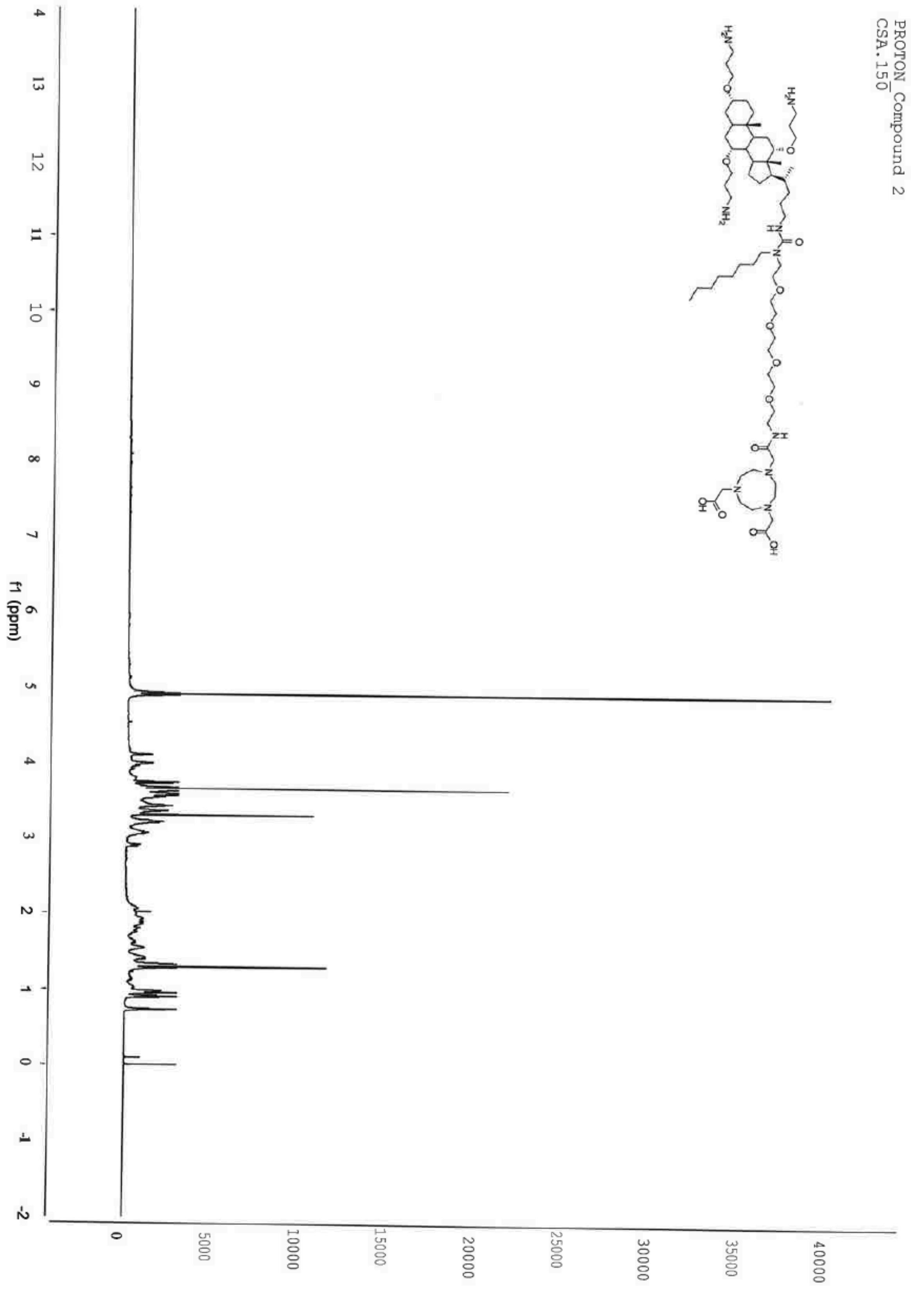
PROTON_Compound 2
CSA.150
f1 (ppm)

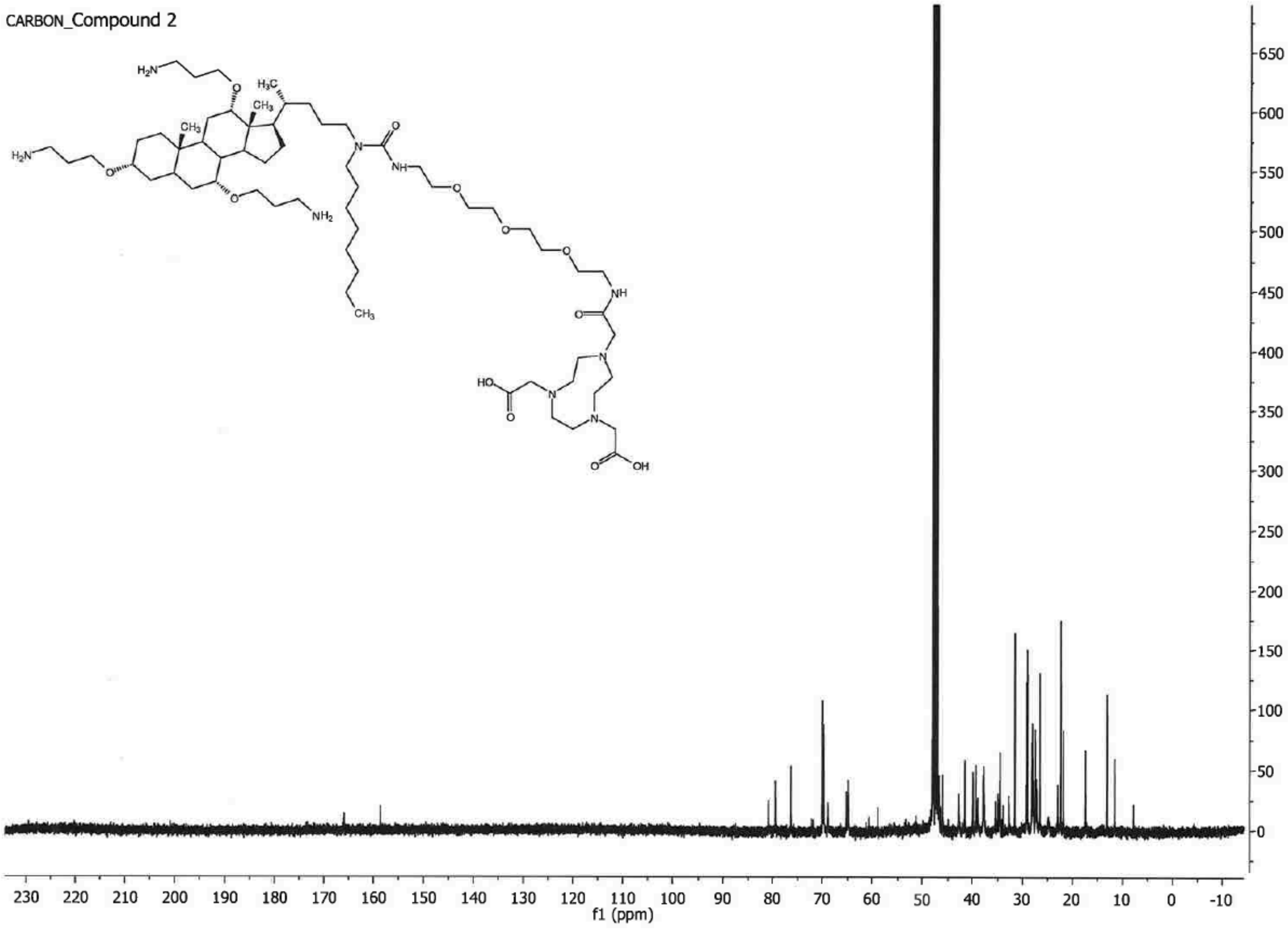

CARBON_Compound 2
f1 (ppm)

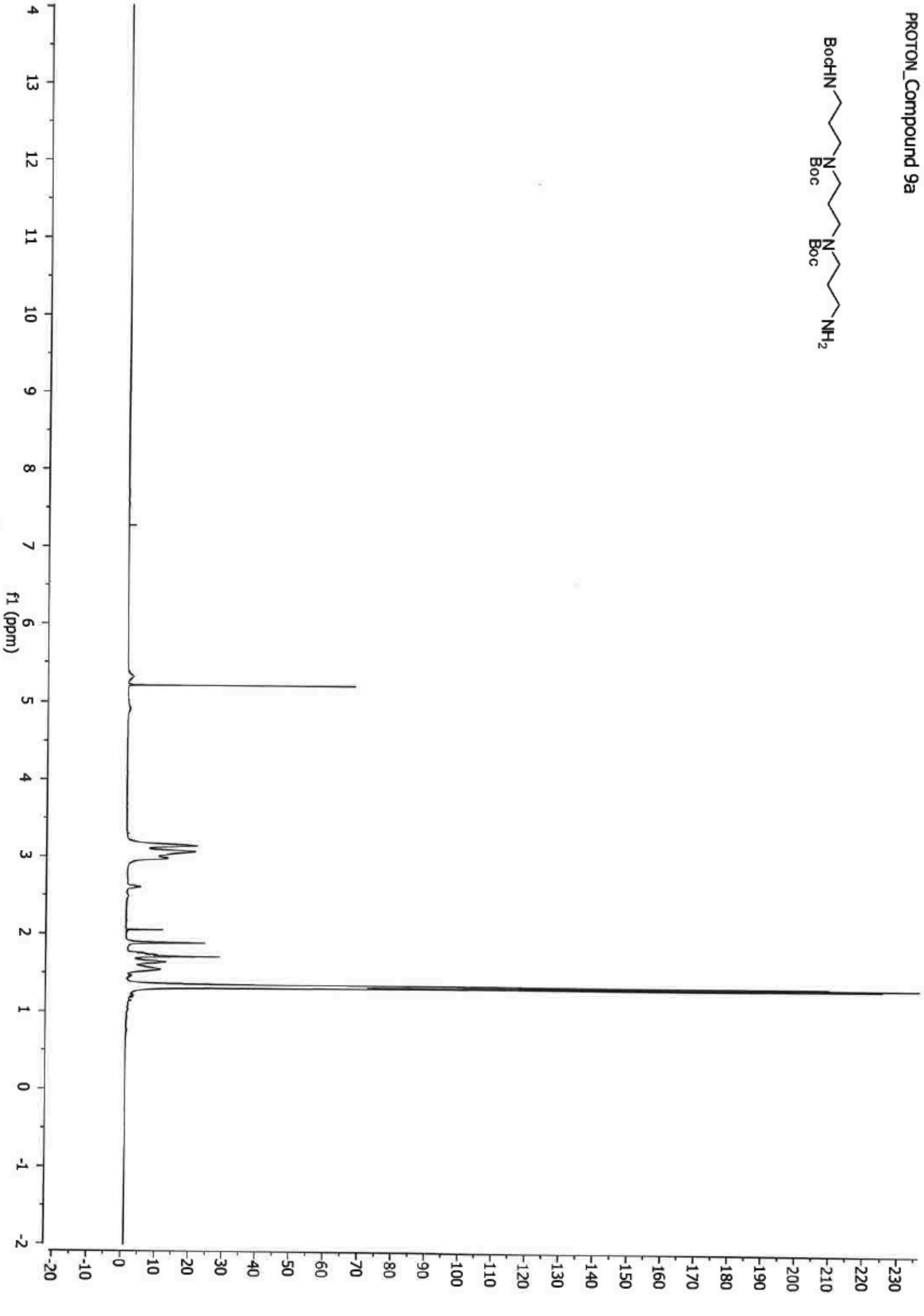
PROTON_Compound 9a
BocHN
N
Boc
N
Boc
NH2
f1 (ppm)

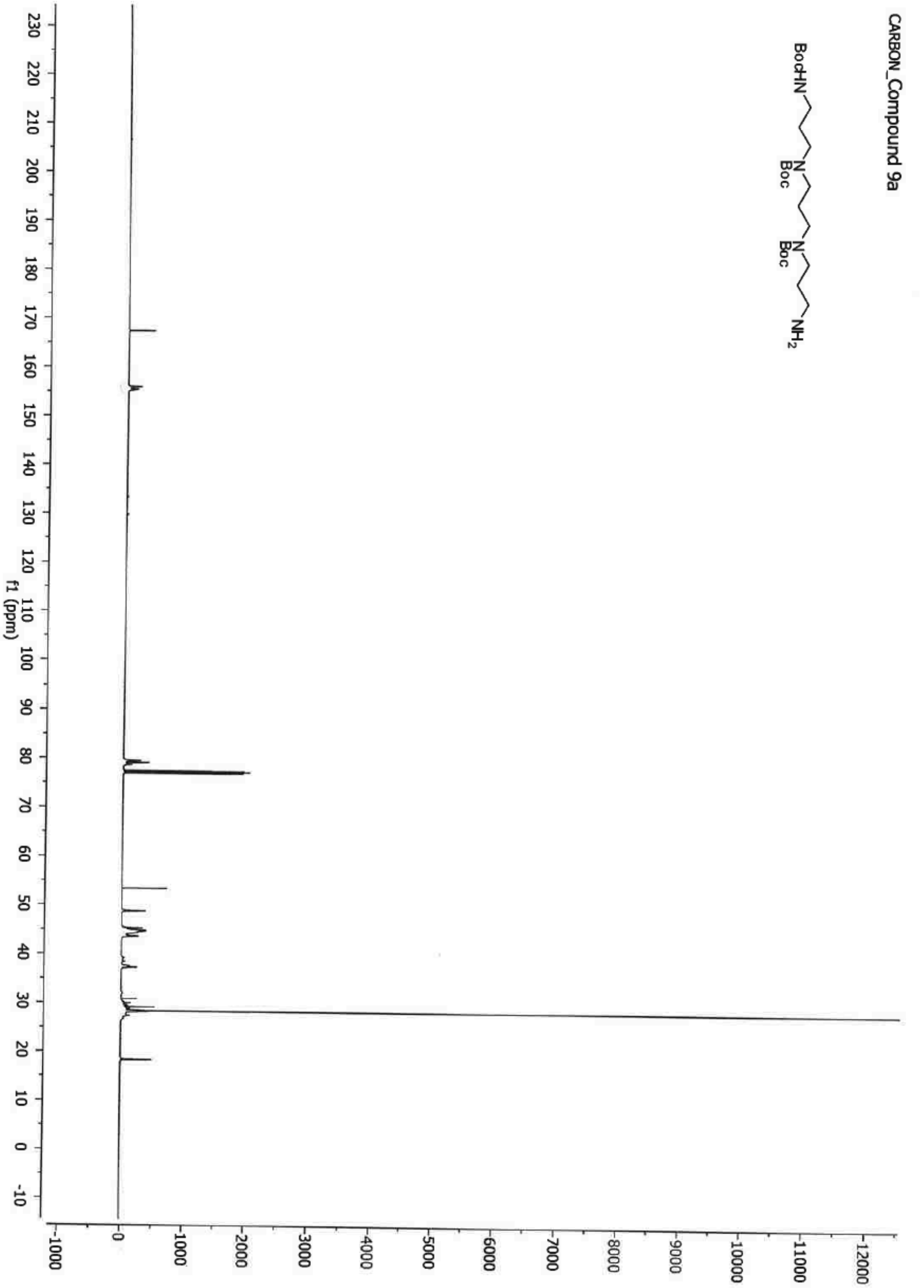
CARBON_Compound 9a
BocHN
N
Boc
N
Boc
NH2
f1 (ppm)

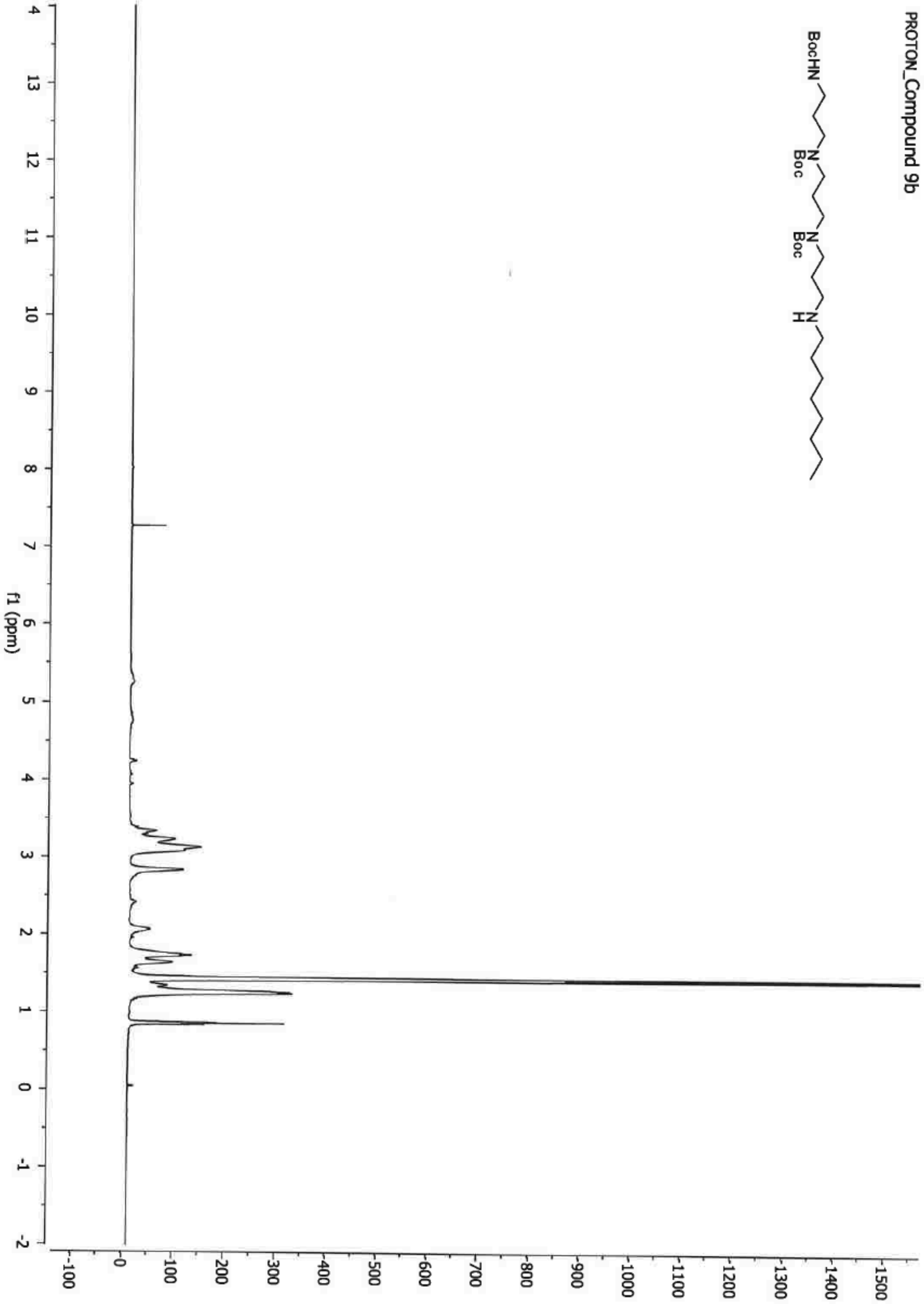

PROTON_Compound 9b
BocHN
N
Boc
N
Boc
N
H
f1 (ppm)

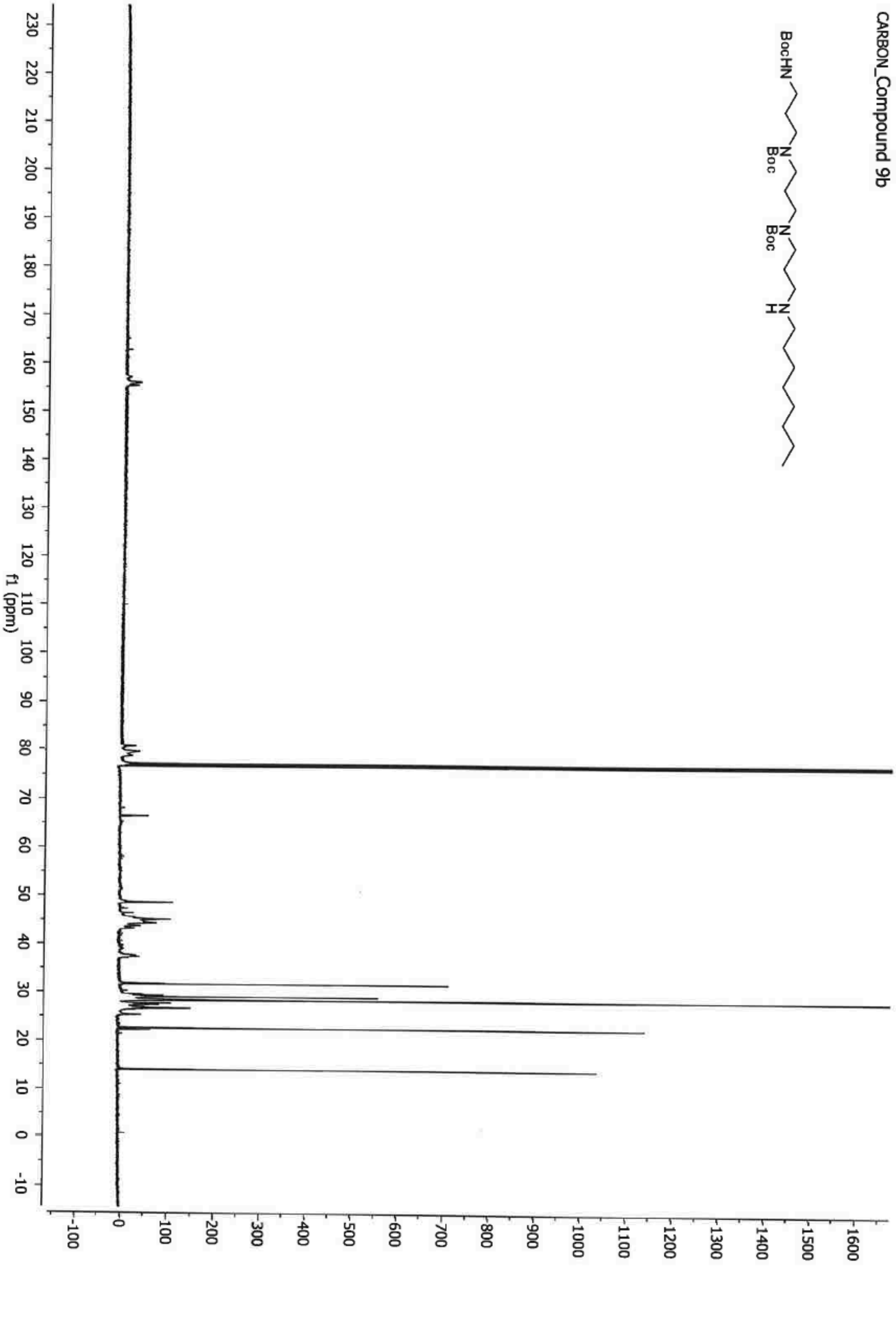
CARBON_Compound 9b
BocHN
N
Boc
N
Boc
N
H
f1 (ppm)

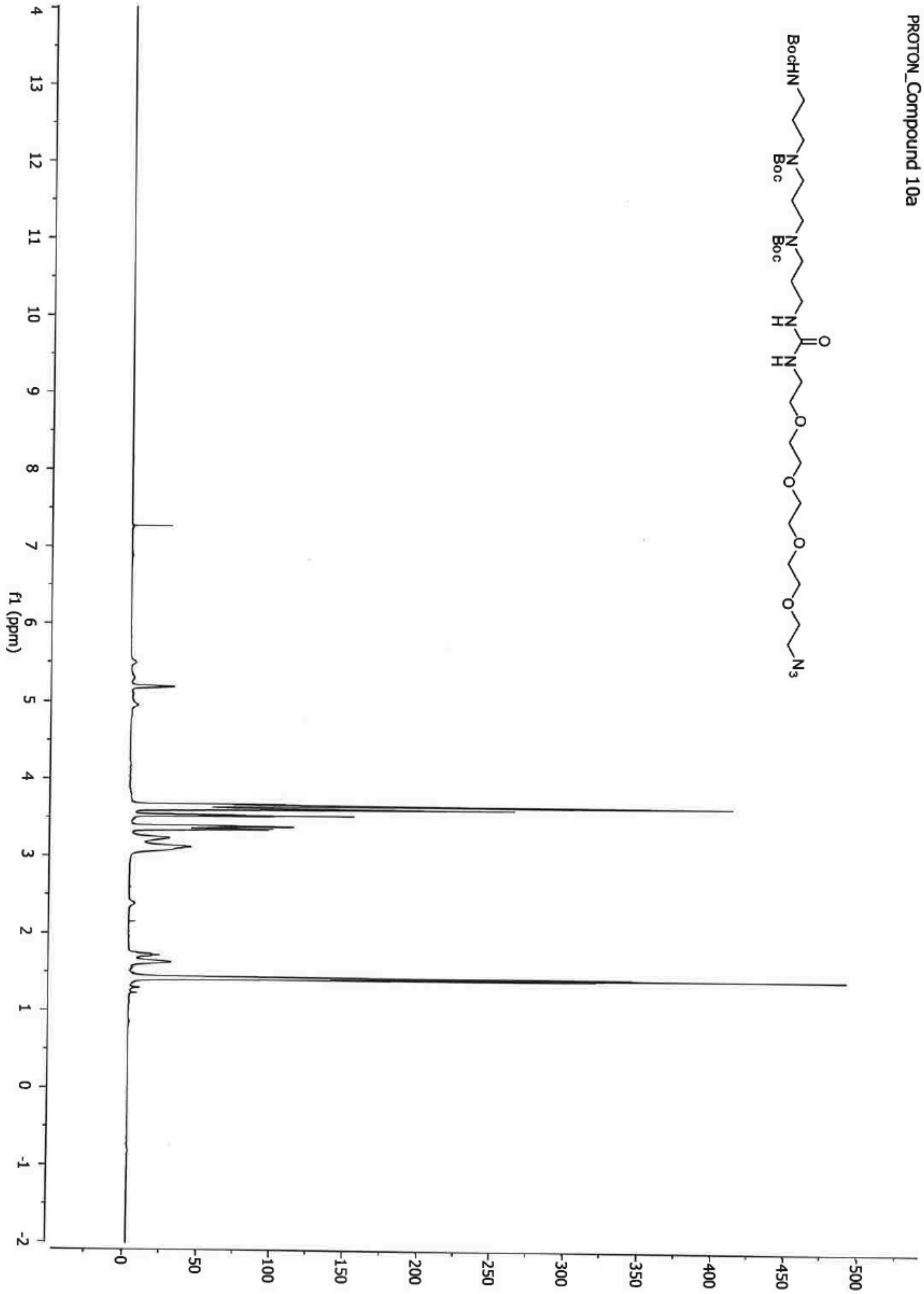
PROTON_Compound 10a
BocHN
N
Boc
N
Boc
N
H
O
N
H
O
O
O
O
N3
f1 (ppm)

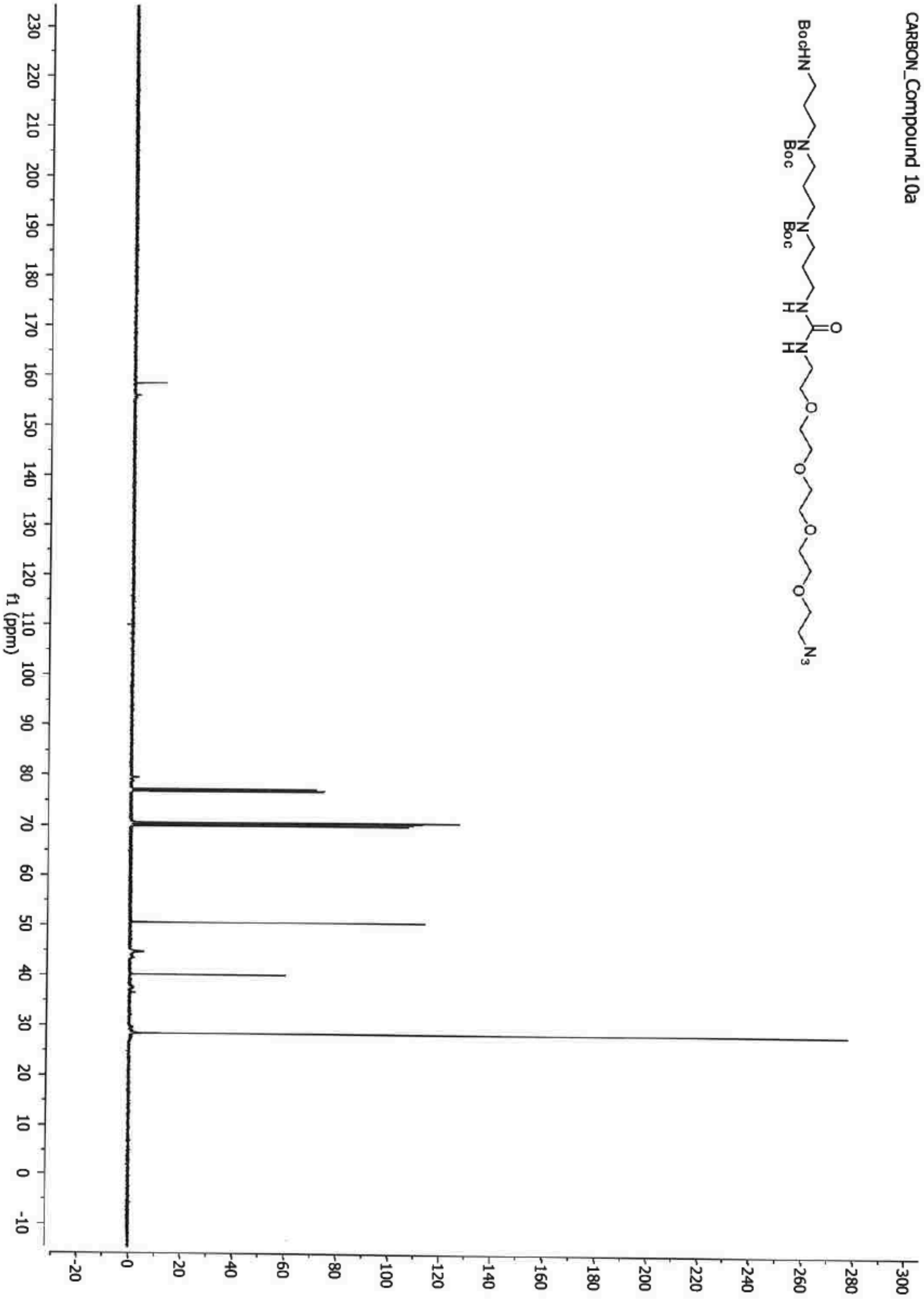
CARBON_Compound 10a
BocHN
Boc
Boc
O
H
H
N
O
N_3
f1 (ppm)

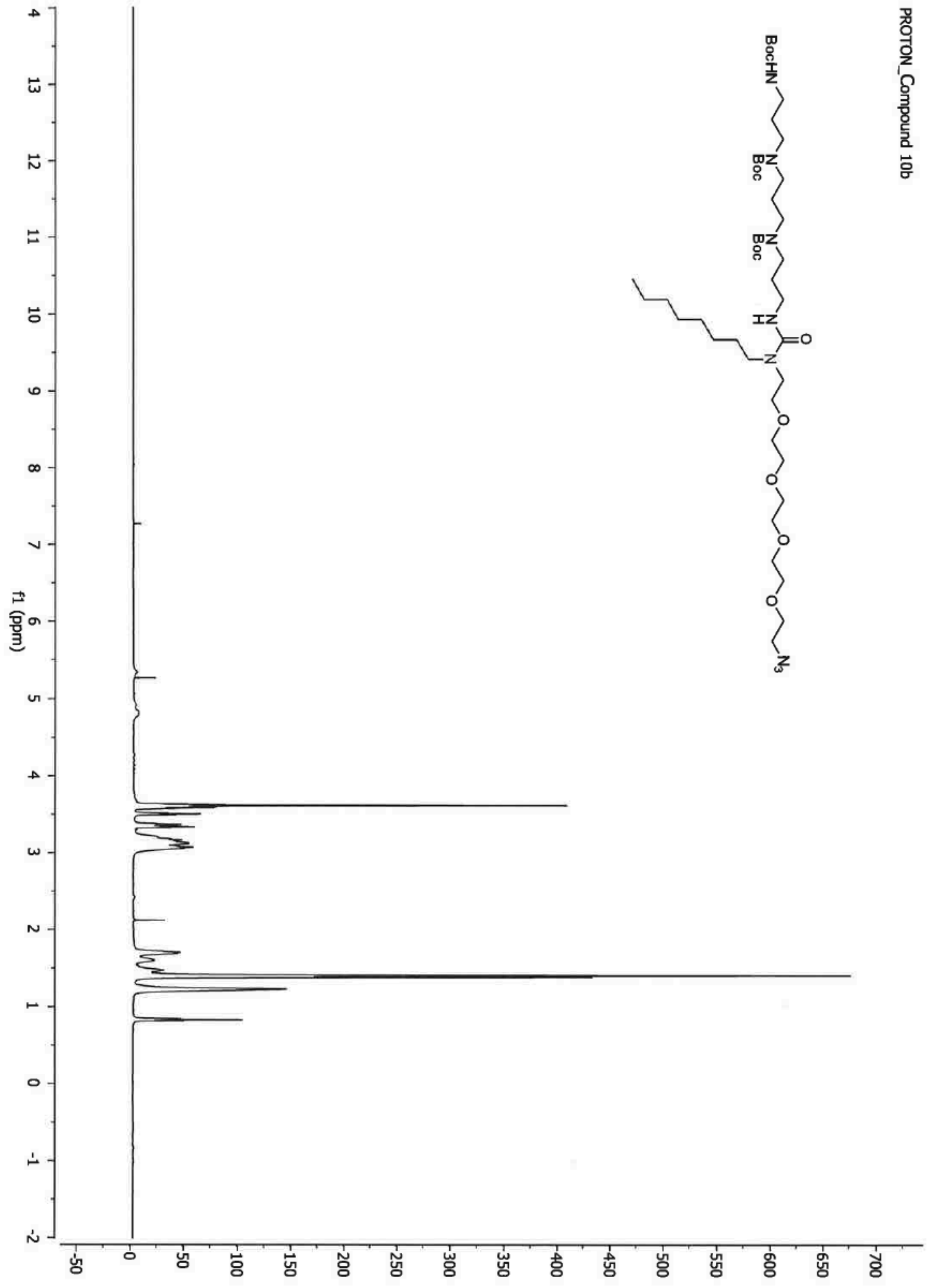
PROTON_Compound 10b
BocHN
N
Boc
N
Boc
N
H
O
N
O
O
O
O
N_3
f1 (ppm)

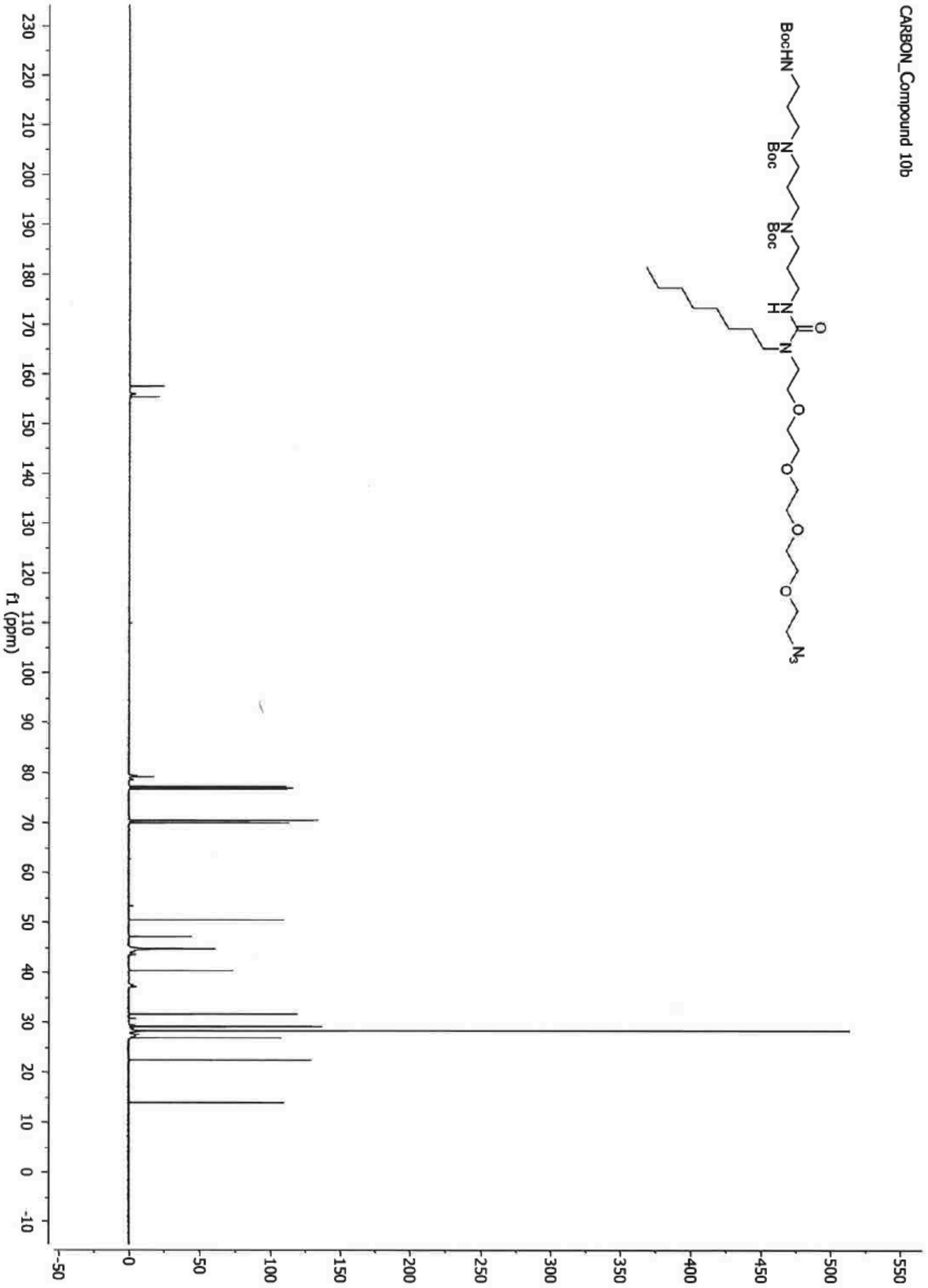

CARBON_Compound 10b
BocHN
N
Boc
N
Boc
N
H
O
N
O
O
O
O
N3
f1 (ppm)
230 220 210 200 190 180 170 160 150 140 130 120 110 100 90 80 70 60 50 40 30 20 10 0 -10
-50 0 50 100 150 200 250 300 350 400 450 500 550

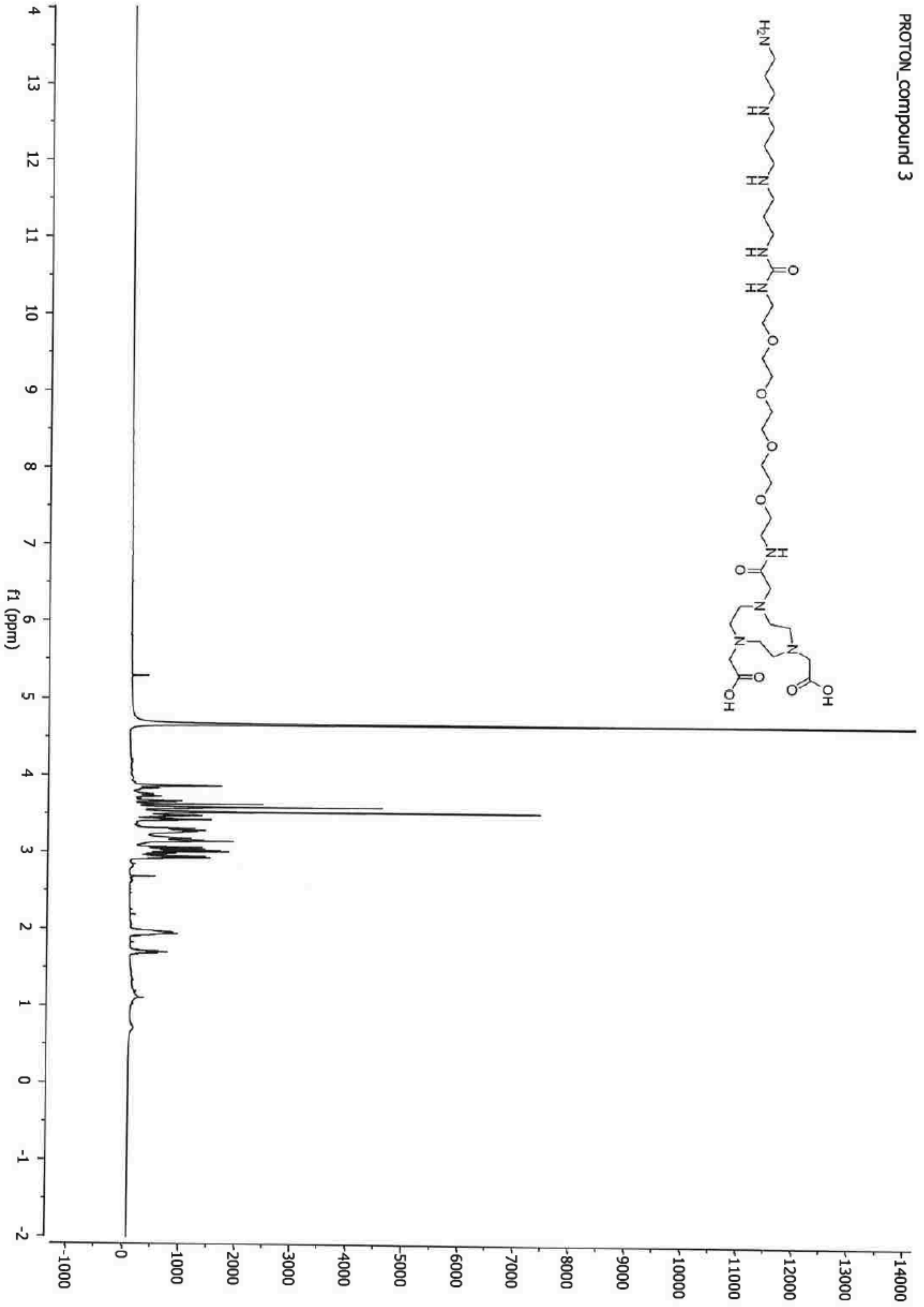
PROTON_compound 3
f1 (ppm)

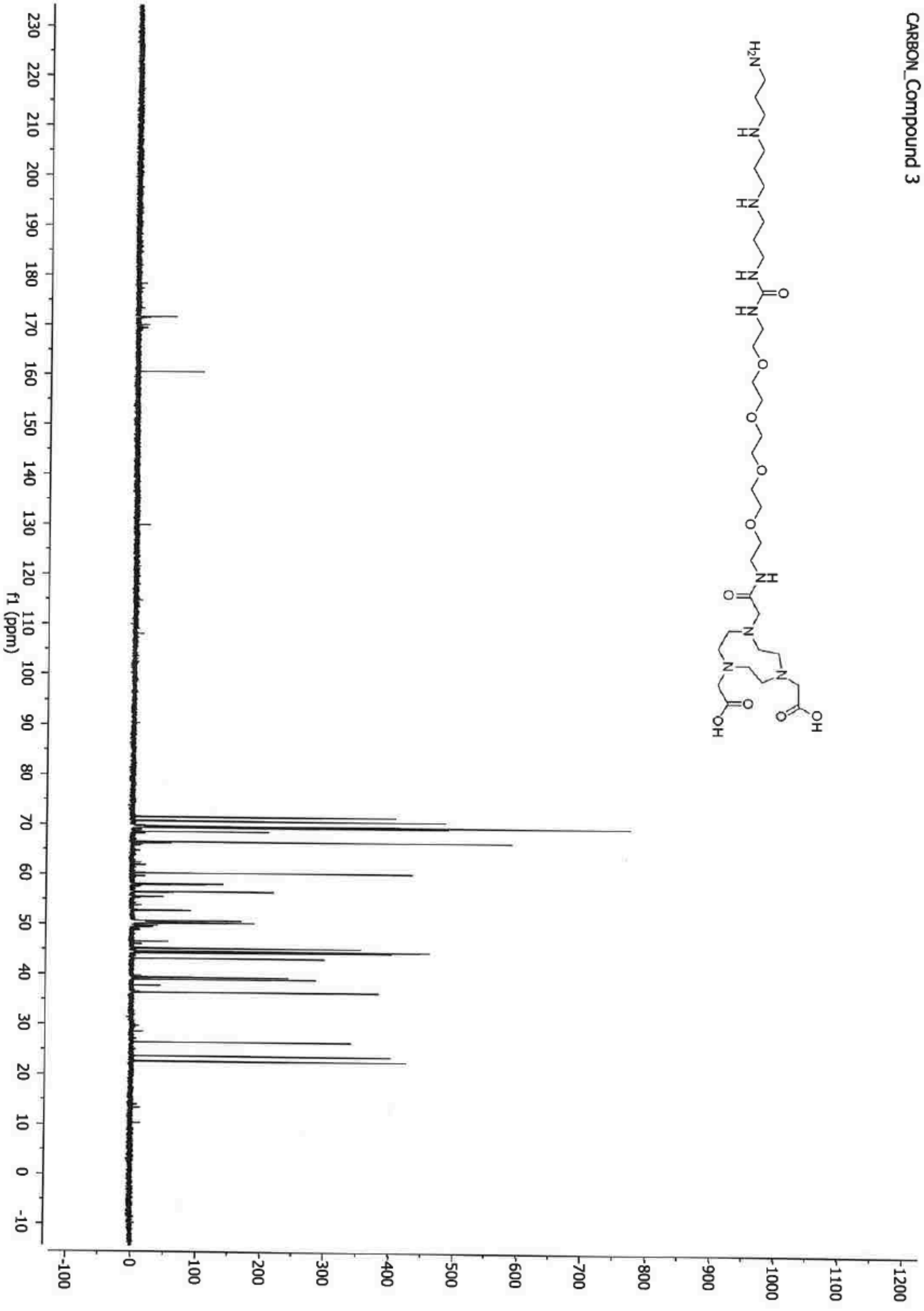
CARBON_Compound 3
f1 (ppm)
230 220 210 200 190 180 170 160 150 140 130 120 110 100 90 80 70 60 50 40 30 20 10 0 -10
-100 0 100 200 300 400 500 600 700 800 900 1000 1100 1200

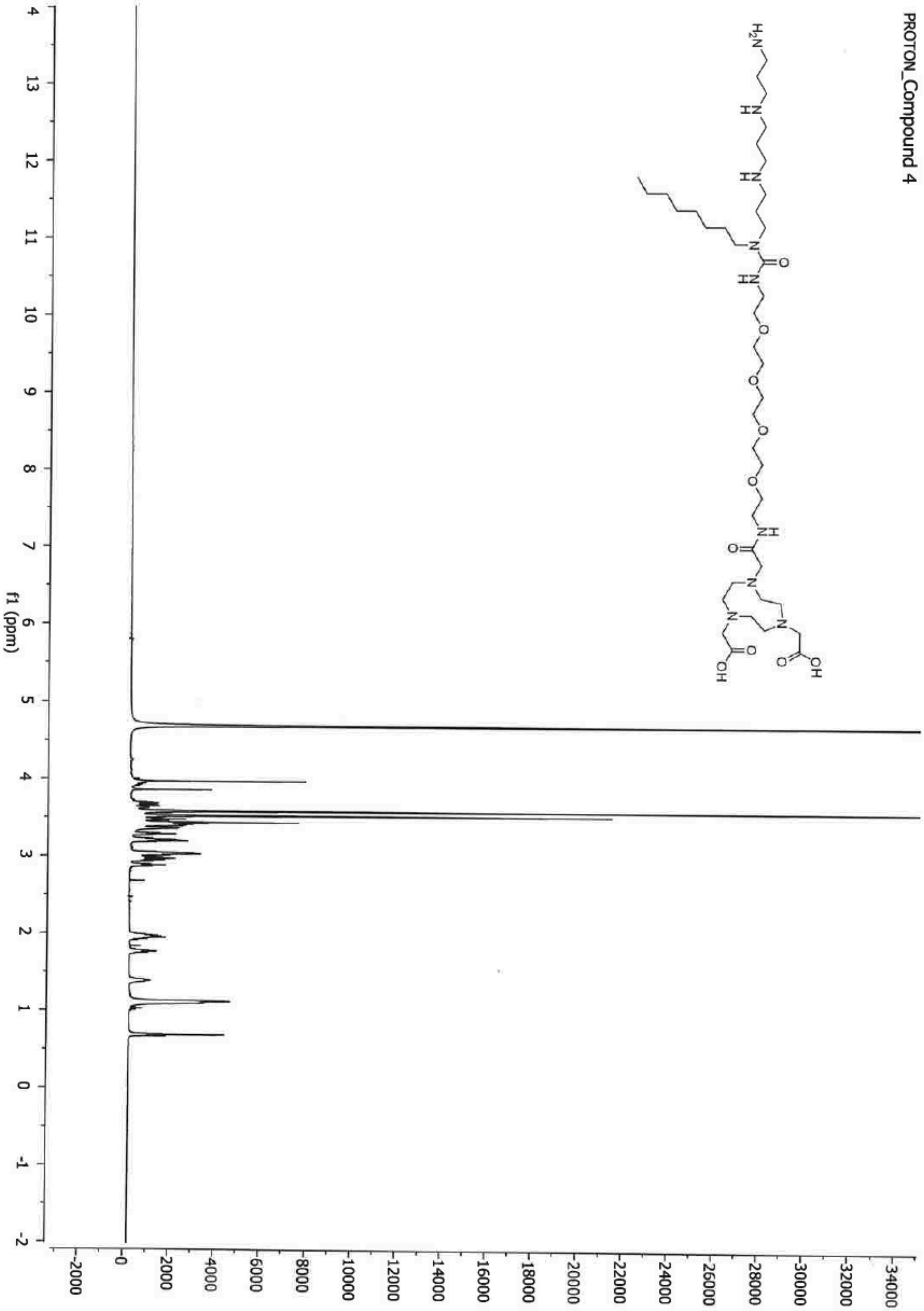
PROTON_Compound 4
f1 (ppm)
14 13 12 11 10 9 8 7 6 5 4 3 2 1 0 -1 -2
-2000 0 2000 4000 6000 8000 10000 12000 14000 16000 18000 20000 22000 24000 26000 28000 30000 32000 34000

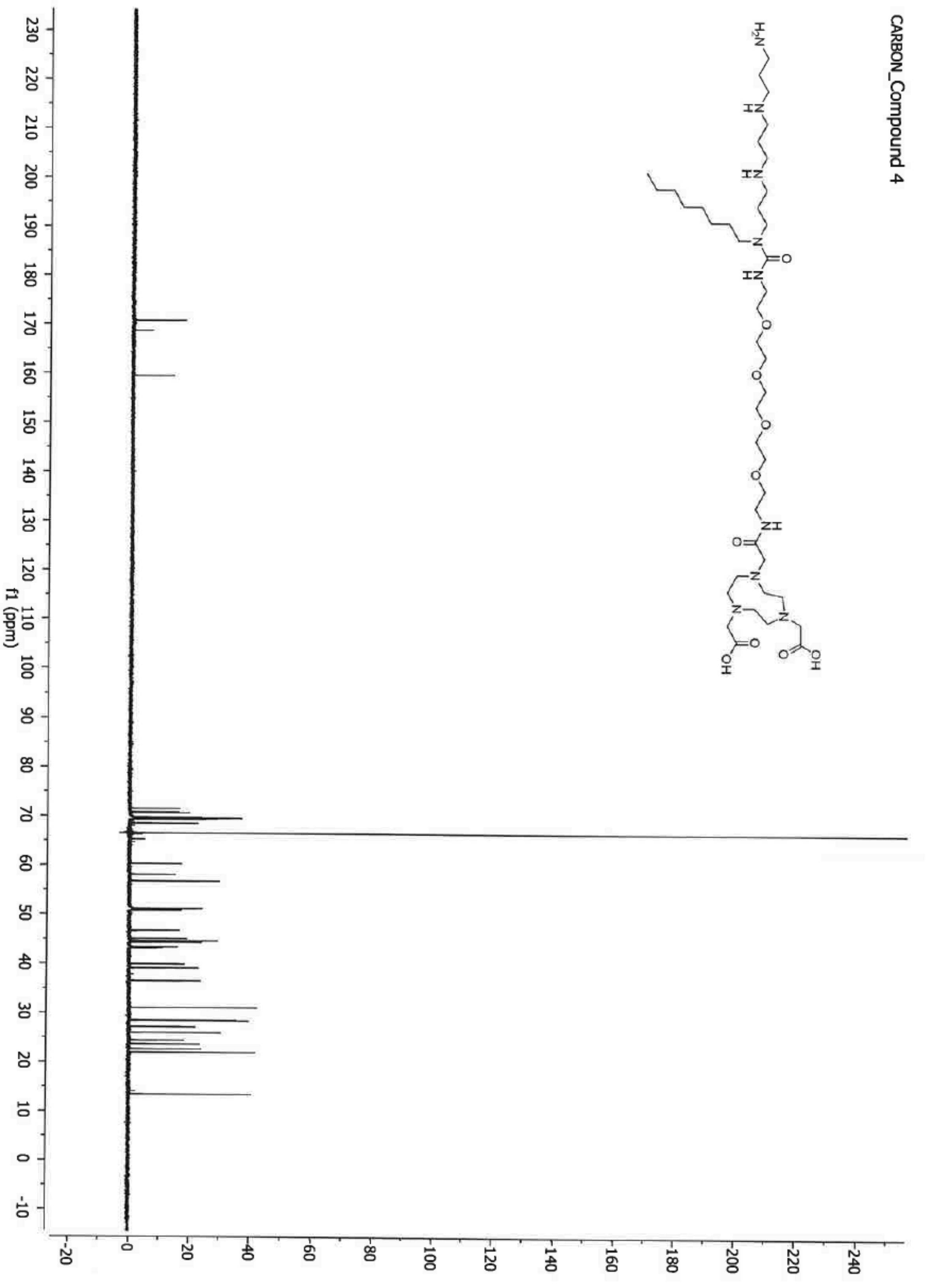
CARBON_Compound 4
f1 (ppm)

PROTON_Compound 6

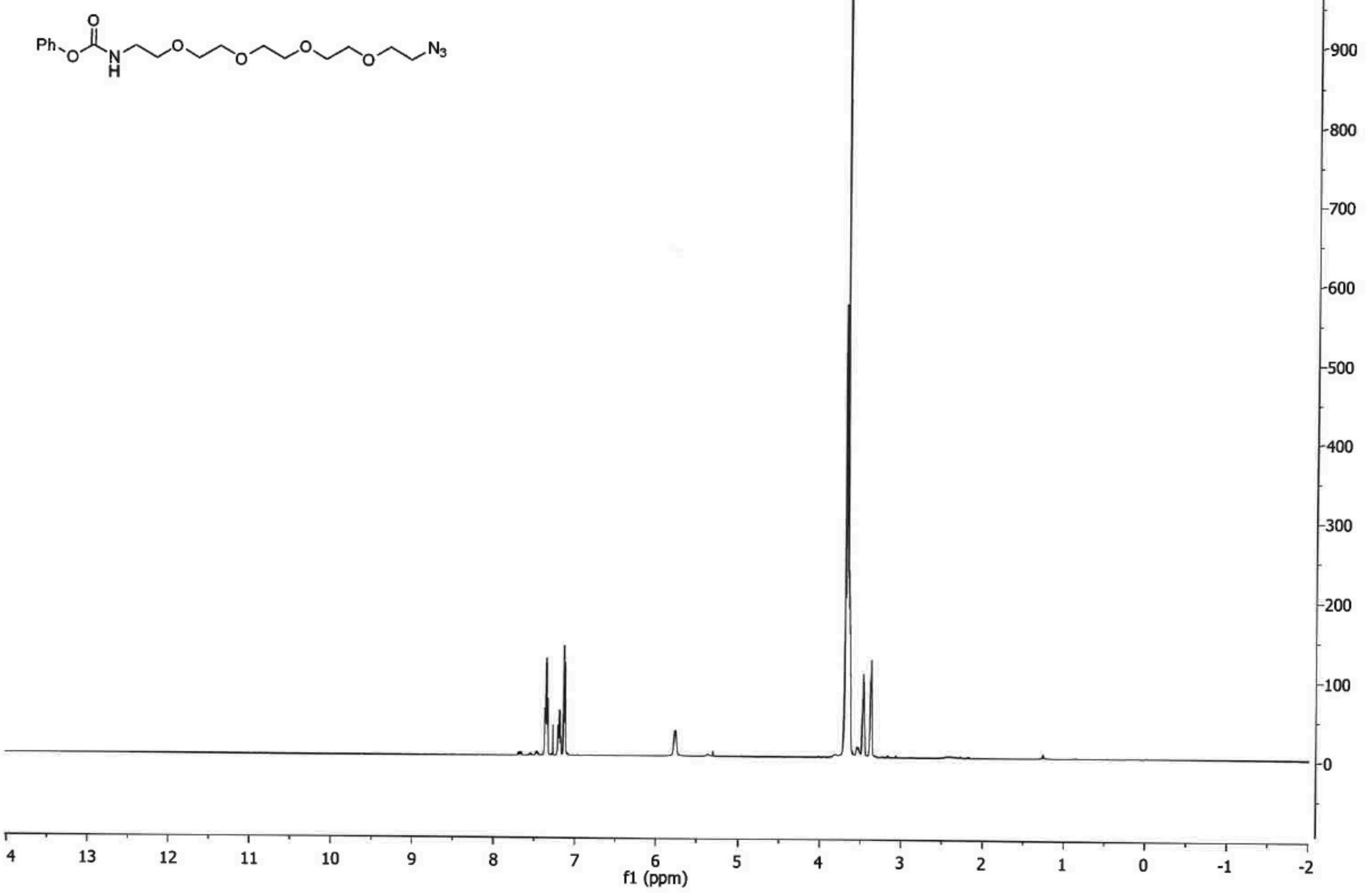

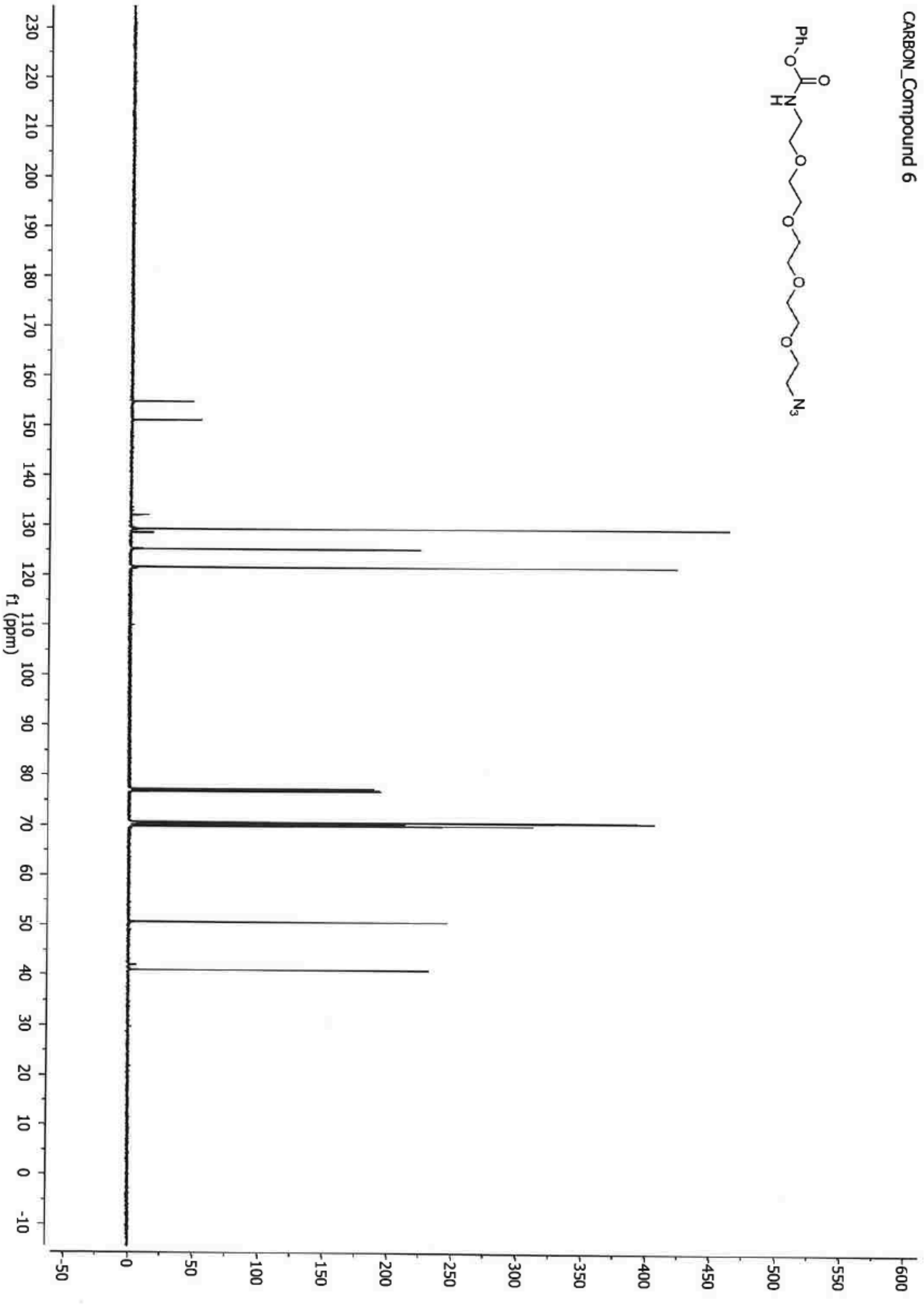
CARBON_Compound 6
f1 (ppm)
